# Intermittent time-restricted feeding recapitulates the physiological benefits of daily time-restricted feeding in male mice with chronic metabolic disease

**DOI:** 10.1016/j.molmet.2026.102432

**Published:** 2026-08-25

**Authors:** Andre Sarmento-Cabral, Marta G. Novelle, Luz Marina Sánchez-Mendoza, Natalia Herman-Sanchez, Samanta Lozano de la Haba, Marta Rodriguez-Vazquez, Pablo Iturbe, Eduardo Chicano-Gálvez, Francisco Javier Cubero, Raul M. Luque, Jose Cordoba-Chacon, Juan L. Lopez-Canovas, Jose M. Villalba, Manuel D. Gahete, Alberto Diaz-Ruiz

**Affiliations:** 1 Department of Cell Biology, Physiology and Immunology, University of Córdoba, Córdoba, Spain; 2 Maimonides Biomedical Research Institute of Córdoba (IMIBIC), Spain; 3 Reina Sofía University Hospital, University of Córdoba, 14004 Córdoba, Spain; 4 Department of Genetics, Physiology and Microbiology (Unity of Animal Physiology), Faculty of Biological Sciences, Complutense University of Madrid (UCM), Madrid, Spain; 5 Laboratory of Cellular and Molecular Gerontology, Precision Nutrition and Aging, Institute IMDEA Nutrition, CEI UAM+CSIC, 28049 Madrid, Spain; 6 IMIBIC Mass Spectrometry and Molecular Imaging Unit (IMSMI), 14004 Córdoba, Spain; 7 CIBEREHD (Center for Biomedical Network Research in Liver and Digestive Diseases), Instituto de Salud Carlos III, 28029 Madrid, Spain; 8 Department of Immunology, Ophthalmology and ORL, Complutense University School of Medicine, Madrid, Spain; 9 Health Research Institute Gregorio Marañón (IiSGM), Madrid, Spain; 10 CIBER Physiopathology of Obesity and Nutrition (CIBERobn), Madrid, Spain; 11 Department of Medicine, University of Illinois Chicago, Chicago, IL, USA

**Keywords:** Time-restricted feeding, Whole-body physiology, Metabolic homeostasis, Lipidomics

## Abstract

Time-restricted feeding (TRF) is proposed as a relevant strategy to counteract obesity and metabolic disorders; however, the therapeutic efficacy of more pragmatic intermittent TRF (iTRF) regimens remains undefined. Herein, daily and intermittent TRF significantly improved whole-body physiology, promoted key hallmarks of energy restriction, and induced coordinated transcriptional and lipidomic remodeling in male mice with chronic metabolic dysfunction previously established by prolonged high-fat, high-cholesterol, high-fructose feeding. These effects were summarized using three composite scores capturing physiological/metabolic, energy restriction, and lipidic responses, which consistently separated daily and intermittent TRF regimens from *ad libitum*-fed mice. Lipidomic profiling identified a subset of hepatic lipids associated with systemic health, pointing to lipid remodeling, particularly at the endoplasmic reticulum, as a potential feature of the TRF response. Notably, TRF failed to histologically improve hepatic steatosis, ballooning, or inflammation, and its overall benefits were clearly inferior to dietary normalization to standard chow diet. Altogether, these data (i) demonstrate that intermittent TRF recapitulates the beneficial effects in advanced metabolic disease, (ii) provides translational support for flexible TRF strategies, and (iii) highlights the need to integrate TRF with additional therapeutic strategies.

## Highlights

• iTRF recapitulates the benefitial effects of dTRF in chronic metabolic disease.
• iTRF and dTRF promote a distinct metabolic state characterized by energy restriction hallmarks and lipid remodeling of the ER.
• TRF variants are not sufficient to resolve advanced hepatic steatosis and inflammation.
• Benefits of TRF schedules reach a physiological plateau and are notably inferior to complete dietary normalization to standard diet.

## 1 Introduction

Noncommunicable diseases (NCDs) account for approximately 75% of non-pandemic-related death worldwide, remaining the leading cause of global morbidity and mortality [[1], [2], [3]]. Overweight and obesity, elevated blood pressure (including hypertension), high blood glucose levels, and abnormal blood lipids (including high cholesterol) represent metabolic risk factors increasing the risk of developing NCDs [1,4]. In 2030, one in two adults will have obesity [1]. In parallel, 25% of the global population is already affected by metabolic dysfunction-associated steatotic liver disease (MASLD), recognized as the predominant cause of liver-related morbidity and mortality [[5], [6], [7], [8]]. Both conditions, obesity and MASLD, constitute chronic, multi-systemic disorders that compromise specific organs beyond adipose tissue and the liver, notably increasing the risk of several co-morbidities such as cardiovascular disease, chronic kidney disease and type 2 diabetes mellitus (T2DM) [9]. The prevalence of MASLD is markedly higher in individuals with obesity and/or T2DM, reaching 70–75% and 80%, respectively [10], in line with the fact that NCDs are interconnected and driven by similar metabolic risk factors. In addition, given that (i) NCDs are complex with no “one-size-fits-all” drug available [11], and (ii) patient adherence to prescribed treatments for NCDs remains suboptimal [12], integrative multidisciplinary approaches in which personalized medication is combined with nutritional-based lifestyle interventions are fundamental to cooperatively reverse interconnected NCDs or to prevent their pathological progression [13,14].

Therapeutic diets based on energy restriction represent sustainable treatments against obesity and MASLD [[15], [16], [17], [18]]. The metabolic response promoted by energy restriction is influenced by fundamental components of the eating architecture, which include timing and distribution of daily calories, meal frequency, and/or the length of inter-meal fasts [19,20]. Beyond the classical continuous caloric restriction, several intermittent energy restriction-based interventions have emerged, which include time-restricted feeding, alternate days of energy restriction, periodic fasting and/or fasting-mimicking diets, the latter designed to induce a physiological state of fasting without complete food deprivation [21]. Shared health-related benefits by energy restriction interventions have been consistently observed in both preclinical and clinical research, and include weight loss, improvement of insulin sensitivity, reduction of chronic inflammation, prevention of cardiovascular and neurological diseases, as well as anti-cancer protection [17,[22], [23], [24]]. Mechanistically, energy restriction triggers the activation of AMP-activated protein kinase, inhibition of the mechanistic target of rapamycin pathway, enhanced autophagy, improved mitochondrial biogenesis and reduced anabolic signaling, all of them considered energy restriction hallmarks. Energy restriction also promotes favorable shifts in gene expression via the modulation of sirtuins (e.g., SIRT1, SIRT3), which influence chromatin remodeling, DNA repair, and metabolic regulation. [25]. Furthermore, energy restriction reshapes the gut microbiome, with significant impact on overall systemic health [26]. These adaptations consistently improve cellular, molecular, metabolic, and physiological homeostasis, altogether slowing the pace of aging and protecting against the appearance and severity of chronic diseases [22].

Among intermittent energy restriction strategies, time-restricted feeding (TRF) constitutes one of the most widely adopted approaches and exhibits the highest short-to medium-term adherence [27]. TRF restricts food intake to specific hours of the day, without necessarily altering total energy consumption. Despite this apparent simplicity, TRF-associated metabolic outcomes are influenced by several key variables including the circadian alignment of the TRF period [28,29]. Of note, important determinants of TRF efficacy remain unresolved, such as the optimal duration of the eating window [28] and/or the quality and nutritional density of food consumed [27]. Moreover, although daily TRF has been extensively investigated, it remains unclear whether intermittent TRF (iTRF) schedules can reproduce the therapeutic benefits of daily TRF (dTRF), as only a limited number of studies have begun to explore iTRF effectiveness. In *Drosophila*, exposure to a 1:1 iTRF regimen [1 day of TRF (20 h fasting) followed by 1 day of *ad libitum* (AL) recovery] significantly extended lifespan and delayed age-associated functional decline in muscle and gut tissues [30]. In mice, although a 4:3 iTRF protocol [four consecutive TRF days (18 h fasting/day) followed by 3 days of AL feeding] enhanced metabolic resilience, preserved lean mass, and remodeled the skeletal muscle [31], its efficacy relative to daily TRF could not be determined due to the absence of a daily TRF control group. Finally, although a 5:2 iTRF regimen [five TRF days (9 h fasting/day) and two AL days] prevented diet-induced body weight gain and hepatic steatosis when initiated concomitantly with high-fat diet feeding [32], its therapeutic effects in pre-existing obesity remain untested.

To address this gap, we performed a head-to-head comparison of daily and intermittent TRF as therapeutic interventions in mice in which chronic metabolic imbalance had already been established by prolonged high-fat, high-cholesterol and high-fructose feeding before TRF initiation. Our data demonstrate that iTRF recapitulates the beneficial effects of dTRF, with both regimens improving obesity-associated metabolic dysfunction across physiological, cellular, and molecular levels.

## 2 Methods

### 2.1 Animal model, diets and feeding schedules

All experimental procedures were carried out in accordance with applicable guidelines and regulations following the European Regulations for Animal Care under the approval of the University of Cordoba and the Regional Government Research Ethics Committees (approved animal protocols number 15/05/2018/088 and 08/03/2022/033).

**Figure 1:**
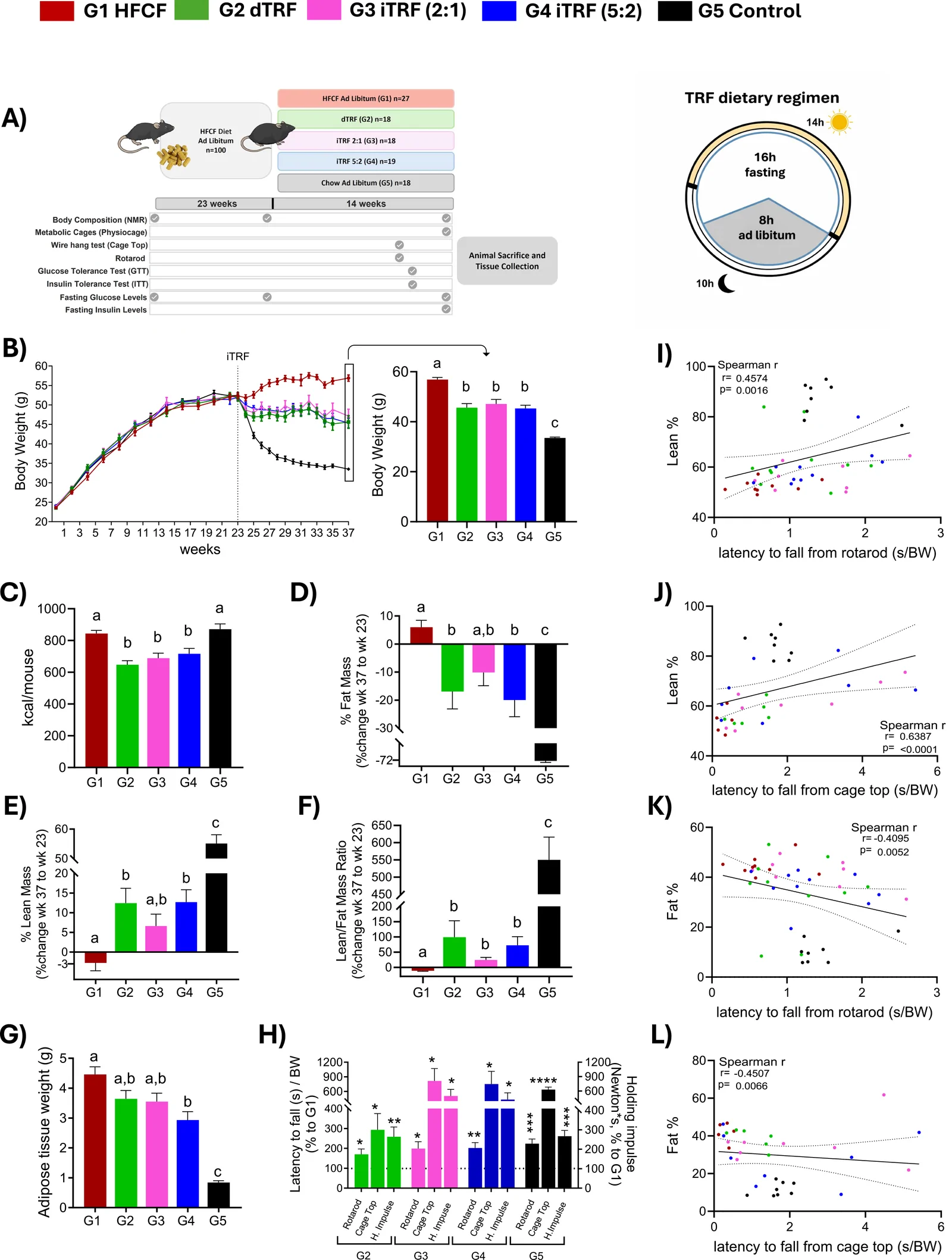
**- Intermittent TRF and daily TRF equally improved physiological health. A)** Mouse models experimental design (left panel), and Light/dark cycles and TRF dietary regime during intervention weeks (right panel). **B)** Body weight evolution during diet induced chronic metabolic imbalance and after iTRF intervention starting at week 23 (dotted line, left panel) and final body weight (week 37, right panel). **C)** Cumulative calorie intake per mouse over the entire intervention. **D-F)** Whole body composition shift after TRF intervention, from week 23 till the end of the study [fat mass percentage **(D)**, lean mass percentage **(E)** and lean to fat mass ratio **(F)**. **G)** sum of the different fat deposits collected (vWAT, sWAT and BAT – visceral, subcutaneous and brown adipose tissue respectively). **H)** Latency time to fall from a rotarod or cage-top, both adjusted by body weight, and holding impulse of cage-top (Newton∗s) of each intervention compared to G1 set at 100% (dotted line). **I-J)** Correlations between whole body lean mass percentage and time to fall from a rotarod **(I)** or cage-top **(J)**. **K-L)** Correlations between whole body fat mass percentage and time to fall from a rotarod **(K)** or cage-top **(L)**. Data are presented as the mean ± SEM. One-way ANOVA tests followed by Tukey's multiple comparation test were implemented to compare data from B, C and G, or Kruskal-Walli's test followed by Dunn's multiple comparation test for panels D, E and F if one or more groups did not follow normal distribution assessed by Shapiro–Wilk test. On panel H a t-test or Mann Whitney test (if one or more groups did not follow normal distribution) comparing each group with HFCF group (set at 100%, dotted line). I-L linear regression with Spearman correlation value (r) and p-value. On B-G, columns that do not share a common letter (a, b, c) are statistically different (p < 0.05). On H, asterisks indicate significant difference (∗p < 0.05; ∗∗p < 0.01; ∗∗∗p < 0.001; ∗∗∗∗p < 0.0001).

Four-weeks-old littermate C57BL/6 male mice were obtained from Charles River Laboratories (Charles River Laboratories, France) and housed under standard conditions for 4 additional weeks until the dietary intervention was initiated as described below. Housing conditions, with sterile filter-capped cages under controlled temperature (22–24 °C) and a modified light–dark cycle (10 h dark/14 h light) remained unchanged throughout the study. To establish a diet induced model of metabolic imbalances, 8 weeks-old C57BL/6 male mice (n = 100) were fed *ad libitum* with a high fat diet with added cholesterol (5.1 kcal/g, 60% kcal fat + 2% cholesterol, D12120101, Research Diets Inc, New Brunswick, NJ, USA) and 10% fructose in drinking water (F0127-5 KG, Merck KGaA, Germany) for 23 weeks (HFCF). Among them, 8 mice did not gain weight and were thus excluded from study. Thereafter, animals were divided into 5 experimental groups depending on the diet and/or the TRF pattern implemented (illustrated in Figure 1A). 1) HFCF group (n = 19), wherein mice were maintained *ad libitum* to the HFCF diet for the duration of the study; 2) daily TRF regimen (dTRF, n = 18), wherein mice were subjected to a daily TRF regimen, consisting of *ad libitum* access to the HFCF diet for 8 h during the dark phase, followed by 16 h of fasting per day with only access to water; 3) intermittent 2:1 TR F regimen (iTRF 2:1, n = 18), wherein mice were subjected to repeated cycles of 2 consecutive days of TRF followed by 1 complete day of *ad libitum* HFCF; 4) intermittent 5:2 TR F regimen (iTRF 5:2, n = 19), wherein mice were subjected to repeated cycles of 5 consecutive days of TRF followed by 2 complete days of *ad libitum* HFCF. Of note, dTRF corresponds to 30 TR F days per month, whereas both iTRF 2:1 and iTRF 5:2 correspond to approximately 20 TR F days per month, representing a ∼33% reduction in TRF days compared with daily TRF. 5) SD group (n = 18), wherein mice were switched to a standard chow diet (3.7 kcal/g, 3.5% kcal fat, LASQCdiet Rod14-R, Altromin Spezialfutter GmbH & COoKG, Germany). Mice were weighed biweekly during the establishment of metabolic imbalance (from week 0 to week 23) and weekly during the TRF intervention, during which food intake was recorded daily.

At the end of the study, animals were sacrificed by decapitation without anesthesia immediately before the initiation of the dark phase, when TRF mice had completed 16 h of fasting, and SD and HFCF mice had completed 14 h of light/inactive phase, a period of low spontaneous feeding activity, although food access was maintained (Supplemental Fig. 1A). Trunk blood was collected in EDTA-containing tubes (BD 363706, Franklin Lakes, NJ, USA) and kept in ice until further centrifugation to obtain plasma. Tissues were immediately excised, weighted and snap-frozen in liquid nitrogen. Plasma and tissues were stored at −20 °C or −80 °C, respectively, until posterior analysis. Additionally, a liver sections were formalin-fixed and paraffin-embedded for histological analysis.

### 2.2 Whole body composition (nuclear magnetic resonance) and indirect calorimetry

Total fat and lean mass were assessed using a Body Composition Analyzer E26-240-RMT (EchoMRI LLC, Houston, TX, USA) at the beginning of the study, after metabolic imbalances establishment, and after TRF intervention. Additionally, whole-body metabolic rate was determined by indirect calorimetry after TRF intervention (before sacrifice), as well as spontaneous activity, using the PHYSIOCAGE system (Harvard, Holliston, MA, USA) and METABOLISM analysis software (Panlab Harvard Apparatus, Barcelona, Spain). Mice were single housed in the metabolic chambers and acclimated to the environment for 48 h prior to data collection. Over five consecutive days, respiratory gases were continuously monitored to quantify oxygen consumption and carbon dioxide production, using the last 3 days for analysis. Gas sampling was performed multiplexed across chambers, utilizing a 2-minute recording interval per cage every 24 min. The resulting respiratory data were used to calculate the Respiratory Exchange Ratio (RER).

### 2.3 Physical performance tests

Physical performance was measured by cage-top (wire hang test) and rotarod (to evaluate motor coordination and balance) after TRF intervention (week 32). Mice underwent a habituation test 3 days prior to the actual test, during which they were placed on the rotarod (LE8505 Panlab) for 1 min at a constant speed (4 rpm). The results represent the average of three rounds per mouse, where the latency time to fall from a rotarod, that accelerated from 4 to 40 rpm over a 5-minute period was measured. Each round had a maximum duration of 5 min, with 15 min resting period between each round. The cage-top test consisted in suspending the animal in an inverted position from wire cage hoppers, positioned one meter above a cushioned surface. Mice were granted the liberty to grip with all four paws and move freely within the hopper, with delicate manual adjustments applied to deter tail entanglement around the wire. Then, we measured the time between the moment of inversion and when animals released the cage hoppers, to calculate the average latency to fall. Between three consecutive rounds, mice were given a 30-minute rest period. The holding impulse was calculated as previously described [33].

### 2.4 Glucose and insulin tolerance tests

The response to glucose or insulin overload was performed after iTRF intervention (week 33). As previously reported [34], glucose tolerance test (GTT; glucose solution 1 g/kg body weight, intraperitoneal injection) was carried out before the initiation of the dark phase, after 16 h of food removal for all groups. Insulin tolerance test (ITT; insulin solution 0.75 U/kg Novolin, intraperitoneal injection) was performed before the initiation of the dark phase. At this point, TRF groups had undergone 16 h of food removal, whereas SD and HFCF control mice had experienced 12 h of the light phase followed by 2 h of food removal. During GTT and ITT, blood glucose levels were accessed by tail vein sampling at 0-, 15-, 30-, 60-, and 120-min, after glucose or insulin injection, using a standard glucometer (GlucoMen areo 2 K; A. Menarini Diagnostics, Firenze, Italia).

### 2.5 Plasma glucose, insulin, leptin and alanine aminotransferase quantification

Fasting glucose was measured after 14 h food removal in tail vein blood sampling at the beginning of the study, after metabolic imbalances establishment, and after iTRF intervention (GlucoMen areo 2 K). Plasma fasting insulin (EZRMI-13 K, EDM Millipore, St. Louis, MO, USA) and leptin (EZML-82 K, EDM Millipore) were measured in a tail vein blood sample after 14 h food removal, one week before killing the animal. Finally, alanine aminotransferase plasma levels (ALT, A7526-150, Pointe Scientific, Canton, MI, USA) were measured from trunk blood samples at the end of the study. All assays were used following manufacturer's instructions. Further details regarding the protocol, specificity, detectability, and reproducibility for each assay can be accessed at the manufacturer's websites.

### 2.6 Liver triglycerides quantification

Frozen livers samples were weighed (30–35 mg) and transferred into microtubes and lysed in 0.3 mL of chloroform/methanol (2:1 vol/vol) at 25 Hz/2 min (Tissuelyser II, Qiagen). Then, the lysates were homogenized for 3 h at 38rpm rotation, and supernatants were acquired. Afterwards, 0.09 mL of MiliQ Water was added in each tube. Samples were then centrifuged at 13,300 rpm for 20 min at Room temperature and the organic fraction was acquired and dissolved in 0.3 mL of chloroform. Triglycerides were measured with a quantitative-Colorimetric Triglyceride Determination Kit (GPO-POD, 1,001,314, Spinreact) following manufacturer's protocol. Briefly, 15 μL of sample and 135 μL of the specific reagent was dispensed into a 96-well plate and incubated for 5 min at 37 °C, followed by absorbance measurement at 505 nm.

### 2.7 Isolation of mitochondria-enriched fractions

Liver tissue samples were homogenized using a steel ball mill (MM400, Retsch, Germany) for 2 min at 25 strokes/s with pre-chilled adaptors in an isolation buffer composed of 20 mM Tris–HCl pH 7.6, 40 mM KCl, 0.2 M sucrose, 1 mM PMSF, 10 mM EDTA, 1 mM DTT, 20 μg/μL CLAP, and phosphatase inhibitor cocktails 2 and 3 (Sigma–Aldrich) diluted at 1/100. The homogenates were centrifuged at 420×*g* for 10 min to remove debris and cell nuclei, and the supernatants were collected. Supernatants were then centrifuged again at 6,700×*g* for 10 min to pellet a mitochondria-enriched fraction. These pellets were resuspended in 100 μl of isolation buffer and stored at −80 °C for subsequent analysis. Total protein content in both the RIPA extracts and the mitochondria-enriched fractions was determined using the modified Bradford dye-binding method developed by Stoscheck [35].

### 2.8 Immunoblotting gel electrophoresis and western blotting

For Western Blot analysis, 20–30 μg of total protein were mixed with Laemmli buffer, boiled and separated on 10% acrylamide gels, and electrophoretically transferred to Hybond-ECL nitrocellulose membranes. Blots were blocked with 5% nonfat dry milk (w/v) in Tris-buffered saline containing 0.1% Tween-20 (TBS-T), then incubated overnight at 4 °C with primary antibodies, prepared in TBS-T with 5% nonfat dry milk, against SIRT3, HSP10, LC3A/B, VDAC, OxPhos cocktail for the mitochondrial complexes quantification, ATF6, and with OxyBlot protein oxidation detection (see Supplementary Table 1 for antibodies dilutions and catalog numbers). The next day, membranes were incubated for 1 h with HRP-conjugated secondary antibodies prepared in 5% nonfat dry milk in TBS-T. After washing, blots were developed using Clarity Western ECL substrate (1,705,060; Bio-Rad, Hercules, CA, USA) for 5 min. Chemiluminescent signals were captured using the ChemiDoc Imaging System (Bio-Rad) and analyzed with Biorad Image Lab 6 software.

### 2.9 Analysis of oxidative markers

Protein carbonyl levels were determined by 2,4-dinitrophenylhydrazine (DNPH) derivatization. Briefly, two aliquots of 20 μg of protein were prepared; one was used as a negative control, and the other one was treated with DNPH using an OxyBlot kit (Millipore, Billerica, MA). Proteins were separated by immunoblotting as indicated above, and the derivatized products were then detected using an anti DNP antibody, as described by the manufacturer.

### 2.10 Respirometry analysis with isolated mitochondria

The rate of oxygen consumption by mitochondria was assessed by respirometry measurements in a SeaHorse XFe24 flux analyzer (Agilent, Santa Clara, CA, USA) using mitochondrial fractions isolated from previously frozen tissue [36,37]. The analysis included the determination of maximal respiration involving complexes II + III + IV, as well as complex IV-linked maximal respiration, with 5 μg of protein per assay. Isolated mitochondria were resuspended in 50 μl of MAS buffer (70 mM sucrose, 220 mM mannitol, 5 mM KH_2_PO_4_, 5 mM MgCl_2_, 1 mM EGTA, 2 mM HEPES, pH 7.4), placed in 24-well SeaHorse plates, and then centrifuged at 4 °C and 3200×*g* for 20 min in a plate centrifuge (Eppendorf 5810 R, Hamburg, Germany) to sediment the mitochondria. Subsequently, 450 μl of MAS buffer were carefully added to each well. Assessment of maximal respiration involving complexes II + III + IV was based on the oxygen consumption rate that was stimulated by the addition of succinate in the presence of rotenone and then inhibited by antimycin A (final concentrations: 0.65 mM succinate, 0.26 μM rotenone, 0.46 μM antimycin A). Maximal respiration linked to Complex IV alone was determined from the rate of oxygen consumption stimulated by the addition of N,N,N′,N′-tetramethyl-p-phenylenediamine (TMPD) plus ascorbic acid and then inhibited by sodium azide (final concentrations: mixture of 0.5 mM TMPD and 2 mM ascorbic adjusted pH to 7.2 with KOH, 4.6 mM azide [38].

### 2.11 Liver RNA extraction, reverse transcription and dynamic qPCR array based on microfluidic technology for genes expression analysis

RNA extraction and Dynamic qPCR were performed as previously reported by our group [[39], [40], [41]]. In summary, total RNA from liver samples was extracted with TRIzol-Reagent (Thermo-Fisher Scientific, Waltham, MA, USA) following the manufacturer instruction. Then, total RNA concentration and purity were assessed with the Nanodrop-One Microvolume UV–Vis Spectrophotometer (Thermo-Fisher Scientific) prior to retrotranscription. After DNAse treatment (RQ1 RNAse-Free DNAse, #M6101, Promega, Madison, WI, USA), 1 μg of total RNA, was retrotranscribed using a Reverse-Transcription Kit (iScript™ Advanced cDNA Synthesis Kit, 100 × 20 μl rxns #1725038, Biorad)). A qPCR dynamic array based on microfluidic technology (Standard BioTools, #BMK-M-96.96) was implemented to determine the expression levels of key genes belonging to Fatty acid metabolism (43 genes). The specific primers for all mouse transcripts used in this study were specifically designed with the Primer3-software [42,43] and are available at Supplementary Table 2. Liver mRNA expression was normalized using a normalization factor calculated with the expression levels of *Actb*, *Ppia* and *Hprt* using GeNorm 3.3 software, as previously reported [44].

### 2.12 Histopathology and morphology analysis

4% Formalin-fixed paraffin-embedded sections of liver were deparaffinized and stained with hematoxylin and eosin (H&E). Nuclei were counterstained with hematoxylin. Tissues were coded at time of collection to ensure an un-biased analysis; at least 3 images were acquired per tissue section (Bright Field Microscope DM1000, Leica, Wetzlar and ZEISS Axio Lab A1. Carl Zeiss Microscopy GmbH, Jena). Histological scores comprised three histological features, characteristic of MASLD: steatosis (0–3), lobular inflammation (0–3) and hepatocellular ballooning (0–3) performed by two blinded researchers and validated by an experienced pathologist [45].

### 2.13 Liver spatial lipidomics

Liver lipids spatial profiling was performed at the IMIBIC Mass Spectrometry and Molecular Imaging Unit, using MALDI mass spectrometry (MS-Imaging). Briefly, fresh tissue slices were obtained by cutting the samples to a thickness of 10 μm using a cryostat, placed on conductive indium-tin oxide (ITO) slides (Intellislides, Bruker, Germany) and frozen at −80 °C until use.

Then, slides were vacuum-dried for 45 min before sample conditioning. An optimized sample preparation protocol for ITO slides was applied to ensure proper sample handling. Thus, dried samples were submerged in a series of baths containing 100 mM ammonium formate in deionized water to remove sodium and potassium adducts. Subsequently, the slides were vacuum-dried again for 45 min. Then, the matrix was applied over the tissue surface using a solution of norharmane (N6252, Sigma Aldrich, Steinheim, Germany) at 7 mg/mL in chloroform: methanol 66:33% solution. The matrix solution was sprayed in 10 layers at 85 °C with a flow rate of 0.050 mL/min using an HTX TM-Sprayer™ (HTX Technologies, USA). After matrix application, slides were vacuum-dried for 40 min before mass spectrometry acquisition.

Spectral acquisition was performed in positive mode using the timsTOF-FleX hybrid mass spectrometer (Bruker, Germany) along with associated software, including timsControl and FlexImaging (Bruker, Germany). Methods, voltages, laser power parameters, number of shots, pixel size (20 μm), calibration, and other settings were adjusted based on the analytical requirements of the analyses.

### 2.14 Z-score calculations

To analyze overall alterations considering several variables, a metabolic, energy restriction and lipid clusters z-scores were calculated considering different variables. For each variable, z-score was calculated by the difference between a data point and the average of the dataset, then dividing that difference by the standard deviation. Then, z-scores of each variable were summed to generate an overall z-score [46]. Specifically, the following variables for the different z-scores were included: 1) Metabolic z-score: Final body weight, adipose tissue depots [visceral (vWAT), subcutaneous (sWAT) and brown adipose tissue (BAT)], body fat and lean mass percentages, lean-to-fat ratio, fasting leptin and fasting insulin, HOMA-IR (Homeostatic Measure of Insulin Resistance) and QUICKI (Quantitative Insulin Sensitivity Check) indexes; 2) ER z-score included: liver weight, plasma ALT, western blot quantification of mitochondrial complexes I, II, III, IV, and V, VDAC, SIRT3, HSP10, LC3A/B and oxyblot, and mitochondrial maximal respiratory capacity measure by SeaHorse involving complexes II + III + IV and complex IV alone; 3) lipid clusters z-score included the 25 lipids represented on Figure 5B, used to generate the 3 lipid clusters.

Of note, when z-score of HFCF resulted to be higher than SD group, the signal of z-score of the variable was inverted to negative, considering positive z-score as a positive effect of the interventions [e.g. a decrease in body weight (HFCF z-score >SD, converted to –HFCF z-score <SD) and an increase in lean mass percentage (HFCF z-score <SD, no conversion)]. To perform z-score correlations with at least two samples per group, the variable leptin and the variables ALT and liver weight were excluded from the metabolic z-score and the energy restriction z-score.

### 2.15 Statistical analysis and graphics generation

Data are represented as mean +/− standard error of the mean (SEM). For certain endpoints, data from some samples were not obtained due to technical limitations, processing errors, or problems encountered during sample preparation or loading. Before proceeding with data analysis, the Shapiro–Wilk test was first used to evaluate normal distribution patterns. If all groups passed the normality test, a one-way ANOVA analysis was performed followed by the Tukey's multiple comparison test to compare all groups [HFCF, SD, dTRF, iTRF 2:1 and iTRF 5:2]. If one of the groups did not pass the normality test, Kruskal–Wallis's test was performed followed by Dunn's multiple comparison test. Additionally, and depending on the group normality, either a t-test or Mann–Whitney test was used when comparing single groups with HFCF group. Statistical significance was considered when p-value <0.05, while p < 0.1 was indicated as a trend (t). Equivalence among different TRF schedules was assessed using two one-sided tests (TOST), with equivalence bounds defined as +/− standard deviation of the dTRF group for each variable. Effects were considered equivalent when both one-sided p-values were <0.05, and inconclusive when only one of the two one-sided tests reached significance (Supplementary Table 3). Analyses were performed using the “TOSTtow” from the “TOSTER” package for RStudio, using the interface available at https://r-statistics.co/tools/equivalence-noninferiority-calculator.html.

Clustering, heatmaps, and Partial Least Squares-Discriminant Analysis (PLS-DA) were performed using MetaboAnalyst 5.0 (https://www.metaboanalyst.ca). The data matrix was uploaded as a. CSV file for analysis. Prior to multivariate modeling, data preprocessing was conducted within the MetaboAnalyst environment. Missing values were imputed using a K-Nearest Neighbors (KNN) algorithm following a sample-wise approach to minimize bias associated with sparse features. Variables presenting more than 10% missing values across samples were excluded to ensure robustness of downstream analyses. The filtered dataset was subsequently mean-centered and autoscaled (unit variance scaling) to standardize variable contribution before generating clustering, heatmap, and PLS-DA visualizations.

## 3 Results

### 3.1 Both intermittent and daily TRF promote physiological health

To study the effectiveness of several variants of time-restricted feeding (TRF), we conducted a ∼ 9-month study where 8 weeks old C57BL/6 male mice were fed *ad libitum* (AL) for 23 weeks on a metabolic stress-inducing high fat, high cholesterol and high fructose (HFCF) diet. Thereafter, mice were randomized on 5 experimental groups as shown in Figure 1A, left panel: Group 1) HFCF, wherein mice were maintained for another 14 weeks in HFCF conditions; Group 2) daily TRF (dTRF), wherein mice were daily fed under TRF regime (16 h fasting/day; Figure 1A, right panel]; Group 3) intermittent 2:1 cycles of TRF (iTRF 2:1), consisting on repeated cycles of 2 consecutive days of TRF followed by 1 day of AL recovery; Group 4) intermittent 5:2 cycles of TRF (iTRF 5:2), consisting on repeated cycles of 5 consecutive days of TRF and 2 days of AL; and Group 5) SD, wherein mice were switched back to a standard chow diet (SD), thus serving as control for assessing healthy metabolic restoration.

Prolonged exposure of mice to HFCF diet promoted chronic metabolic imbalance as determined by marked (i) body weight gain (Suppl. Fig. 1B; ∼118% gain from baseline) (ii) fat mass increase (∼577%), (iii) lean mass reduction (∼39%) and (iv) reduction of lean-to-fat ratio (∼92%) (Supplemental Fig. 1C–E, respectively). Thereafter, TRF interventions and dietary normalization in SD mice were initiated. As expected, 14 weeks of dietary reversion to a standard diet induced a robust recovery from metabolic disease, evidenced by marked (i) body weight loss (∼36%), (ii) fat mass reduction (∼73%), (iii) lean mass restoration (∼54%) and (iv) increased lean-to-fat ratio (∼545%) (Suppl. Fig. 1F–I, respectively). Remarkably, when compared to HFCF mice, all TRF regimes (daily, iTRF 2:1 and iTRF 5:2) exhibited significant and comparable weight loss (Figure 1B), yet this effect was less pronounced than in SD mice (Figure 1B). Analysis of average food intake revealed that, on TRF days, mice significantly consumed lower calories per day vs. HFCF mice (reduction of ∼23%, ∼41% and ∼32% for TRF, iTRF 2:1 and iTRF 5:2, respectively; Supplemental Fig. 2A). Over the duration of the study, returning to AL conditions in mice subjected to iTRF 2:1 and iTRF 5:2 caused an overeating reaction (Supplemental Fig. 2B–D). Despite this behavior, cumulative calorie consumption per mouse over the entire intervention was reduced for all TRF feeding groups (Figure 1C). Interestingly, percentage body weight gain normalized to cumulative calorie intake remained significantly different between TRF groups and HFCF mice, suggesting that, beyond caloric intake alone, the feeding schedule itself contributes to the observed reduction of body weight (Supplemental Fig. 2E). In addition, similar calorie intake was observed between SD and HFCF mice, likely reflecting lower intake (in grams) of a higher-caloric density diet of HFCF mice (Supplemental Fig. 2F). Interestingly, all TRF regimes promoted equivalent adaptations of body composition, as determined by similar reductions of fat mass concomitant with a similar restoration of lean mass and lean to fat ratio vs. HFCF mice (Figure 1D–F, respectively; and Supplementary Table 4). Consistent with these findings, adipose tissue weights measured at sacrifice tended to be lower across TRF schedules, with no significant differences detected among TRF groups (Figure 1G). Adipose tissue loss was proportionally distributed across wisceral, subcutaneous and brown fat depots (Supplemental Fig. 2G–I, respectively). Likewise, physical performance relative to HFCF mice was enhanced in all TRF variants, evidenced by increased latency to fall from traditional rotarod and inverted cage top tests, together with increased holding impulse as a complementary metric of neuromuscular strength, coordination, and endurance [47,48] (Figure 1H). Performance in both tests correlated positively with lean mass percentage (Figure 1I–J) and negatively with fat mass percentage (Figure 1K–L). Overall, these findings indicate that restricting food intake to an 8-hour window, either daily or in intermittent cycles, constitute effective strategies to promote body weight loss, reduce fat mass, restore lean mass and improve physical performance in mice with established metabolic dysfunction.

### 3.2 Intermittent TRF and daily TRF effectively re-establish metabolic homeostasis

**Figure 2:**
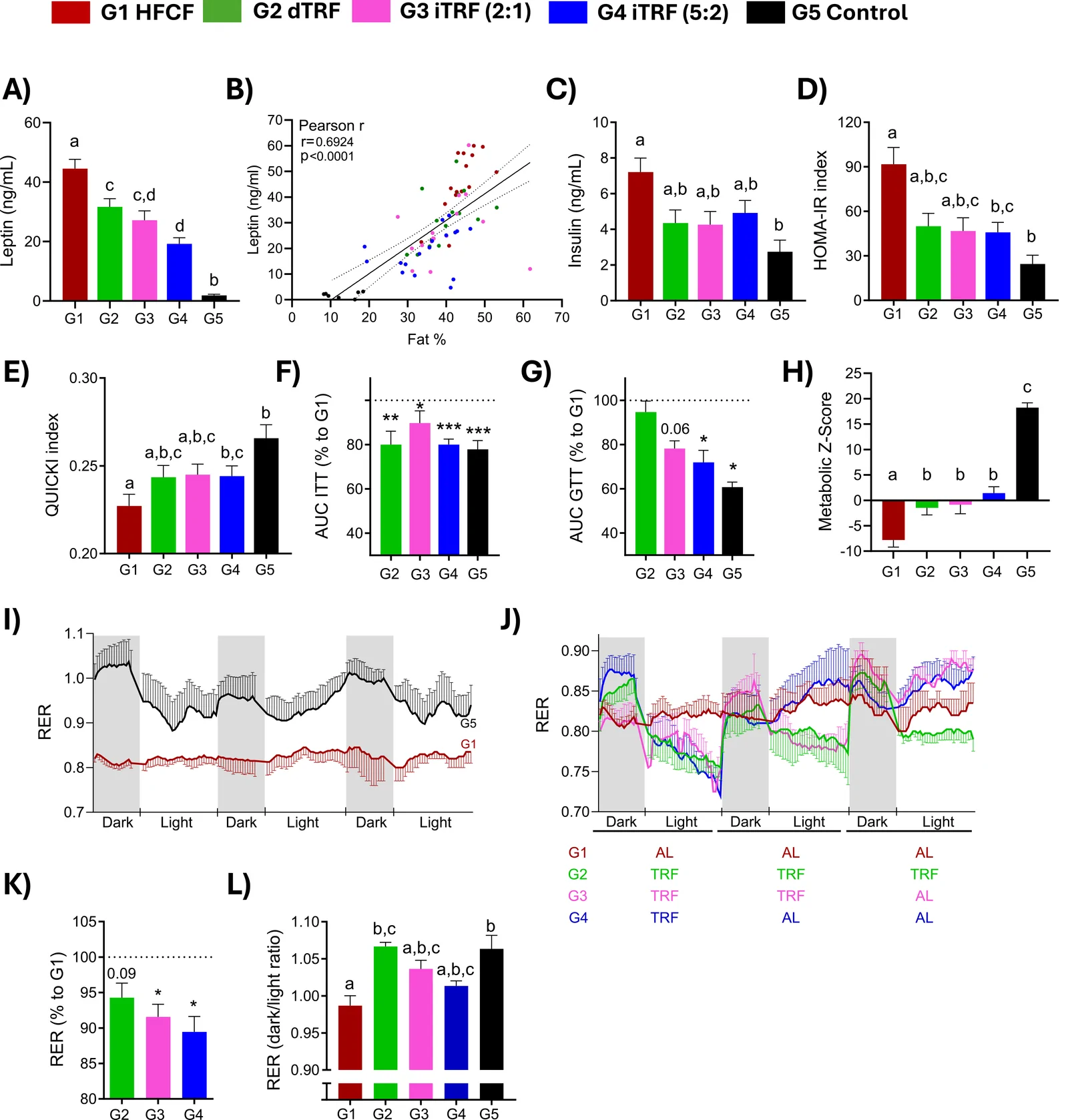
**- Intermittent TRF and Daily TRF equally re-established metabolic homeostasis. A)** Fasting plasma leptin levels after TRF intervention. **B)** Correlation between fasting leptin levels and whole-body fat mass percentage. **C)** Fasting plasma insulin levels after TRF intervention. **D)** Homeostatic Model Assessment for Insulin Resistance index (HOMA-IR). **E)** Quantitative Insulin Sensitivity Check Index (QUICKI). **F-G)** Area under the curve of insulin **(F)** and glucose **(G)** tolerance tests. **H)** Metabolic z-score calculated by the sum of z-scores of the different parameters represented on Figure 1, Figure 2 (Final body weight; visceral, subcutaneous and brown adipose tissue depots; whole body fat and lean mass percentage; lean to fat mass ratio; plasma fasting leptin and insulin; HOMA-IR and QUICKI. **I-J)** Respiratory Exchange Ratio of HFCF and SD groups **(I)**, and HFHC, dTRF, iTRF (2:1 and 5:2) groups **(J)** accessed by indirect calorimetry. **K)** Respiratory Exchange Ratio (RER) average during the last 5 h of light cycle during TRF period [G1 set at 100% (dotted line)]. **L)** Respiratory Exchange Ratio dark/night ratio. Data are presented as the mean ± SEM (A-H, K and L) or average during time (I and J). One-way ANOVA tests followed by Tukey's multiple comparation test were implemented to compare data from A, H and L, or Kruskal-Walli's test followed by Dunn's multiple comparation test for panels C, D and E if one or more groups did not follow normal distribution assessed by Shapiro–Wilk test. On panel F, G and K a t-test or Mann Whitney test (if one or more groups did not follow normal distribution) comparing each group with HFCF group (set at 100%, dotted line). Panel B, linear regression with Pearson correlation value (r) and p-value. On A, C, D, E, H and L, columns that do not share a common letter (a, b, c) are statistically different (p < 0.05). On F, G and K, asterisks indicate significant difference (∗p < 0.05; ∗∗p < 0.01; ∗∗∗p < 0.001; ∗∗∗∗p < 0.0001) and if p-value is between 0.05 and 0.1 the p-value is indicated above the column.

Next, we examined various circulating biomarkers related to metabolic health. When compared to HFCF mice, all TRF regimens (daily, iTRF 2:1 and iTRF 5:2) exhibited significant reduction of leptin levels (Figure 2A), consistent with lower adipose tissue (Figure 1G); yet this reduction was less pronounced than in SD controls (Figure 2A). Leptin levels positively correlated with body fat percentage (Figure 2B) and with adipose tissue weights (Suppl. Fig. 3A–C). Likewise, across all TRF variants, we found a comparable reduction in insulin levels as well as of the HOMA-IR (Figure 2C–D). Accordingly, the QUICKI index was elevated in mice exposed to all TRF regimes (Figure 2E). We then analyzed dynamic glucose management by injecting insulin or glucose intraperitoneally. Interestingly, when compared to HFCF mice, all TRF regimens (daily, iTRF 2:1 and iTRF 5:2) exhibited improved glucose clearance from circulation in response to insulin challenge, by means of lower area under the curve (AUC) values, suggestive of improved insulin sensitivity (Figure 2F). Likewise, glucose disposal during GTT was enhanced in TRF mice, although improvement in dTRF mice did not reach statistical significance (Figure 2G). Full test trajectories are presented in Supplemental Fig. 3D–E. Taken together our data suggests that restricting food intake to an 8-hour window - whether implemented daily or intermittently – enhances systemic glucose regulation, improves insulin sensitivity, and promotes overall metabolic health. Interestingly, we calculated a metabolic Z-score integrating all analyzed parameters by standardizing each value relative to the dataset, indicating its deviation from the mean. As shown in Figure 2H, SD mice displayed the highest score compared with HFCF mice. Remarkably, all TRF regimes led to a significant elevation of this score, with no differences detected between TRF groups, supporting the notion that intermittent TRF recapitulates the physiological benefits observed with daily TRF (Figure 2H).

Whole-body energy metabolism was then evaluated. To this end, mice were placed on the PHYSIOCAGE system metabolic chambers, during which O_2_ consumption and CO_2_ production were recorded. Data for mice subjected to cycles of TRF were collected during both TRF and AL days of the cycle. Analysis of the Respiratory Exchange Ratio (RER, VCO_2_/VO_2_ ratio) in SD mice showed well-characterized circadian oscillations between the dark (active) and light (inactive) phases (Figure 2I, black line). In sharp contrast, HFCF mice displayed metabolic inflexibility, characterized by the absence of RER oscillations (Figure 2I, red line). For all TRF feeding groups, food removal during TRF days led to a progressive reduction of RER values during the resting phase vs. HFCF- mice (Figure 2J–K, and Suppl. Fig. 4A), indicating a greater reliance on lipids as energy substrates. In these mice, reintroduction of food following the fasting period led to a modest rebound in RER values, suggestive of a metabolic switch from lipids to carbohydrate utilization (Figure 2J and Suppl. Fig. 4B). In concordance, a higher dark-to-light ratio for RER values was found for all TRF interventions vs. HFCF mice (Figure 2L). Overall, this data indicates that TRF interventions, regardless of whether they are implemented daily or intermittently, effectively influence metabolic flexibility and homeostasis.

### 3.3 Both iTRF and dTRF trigger molecular aspects of energy-restriction

Energy restriction is known to promote a well-characterized set of cellular and molecular responses [22,49]. In this study, we evaluated how daily and intermittent TRF impact hepatic signatures of energy restriction, which included mitochondrial remodeling, attenuation of oxidative stress, sirtuin activation, augmented autophagy and an integrated evaluation of lipid metabolism by combining the analysis of the expression of targeted gene expression and spatial lipidomic profiling.

**Figure 3:**
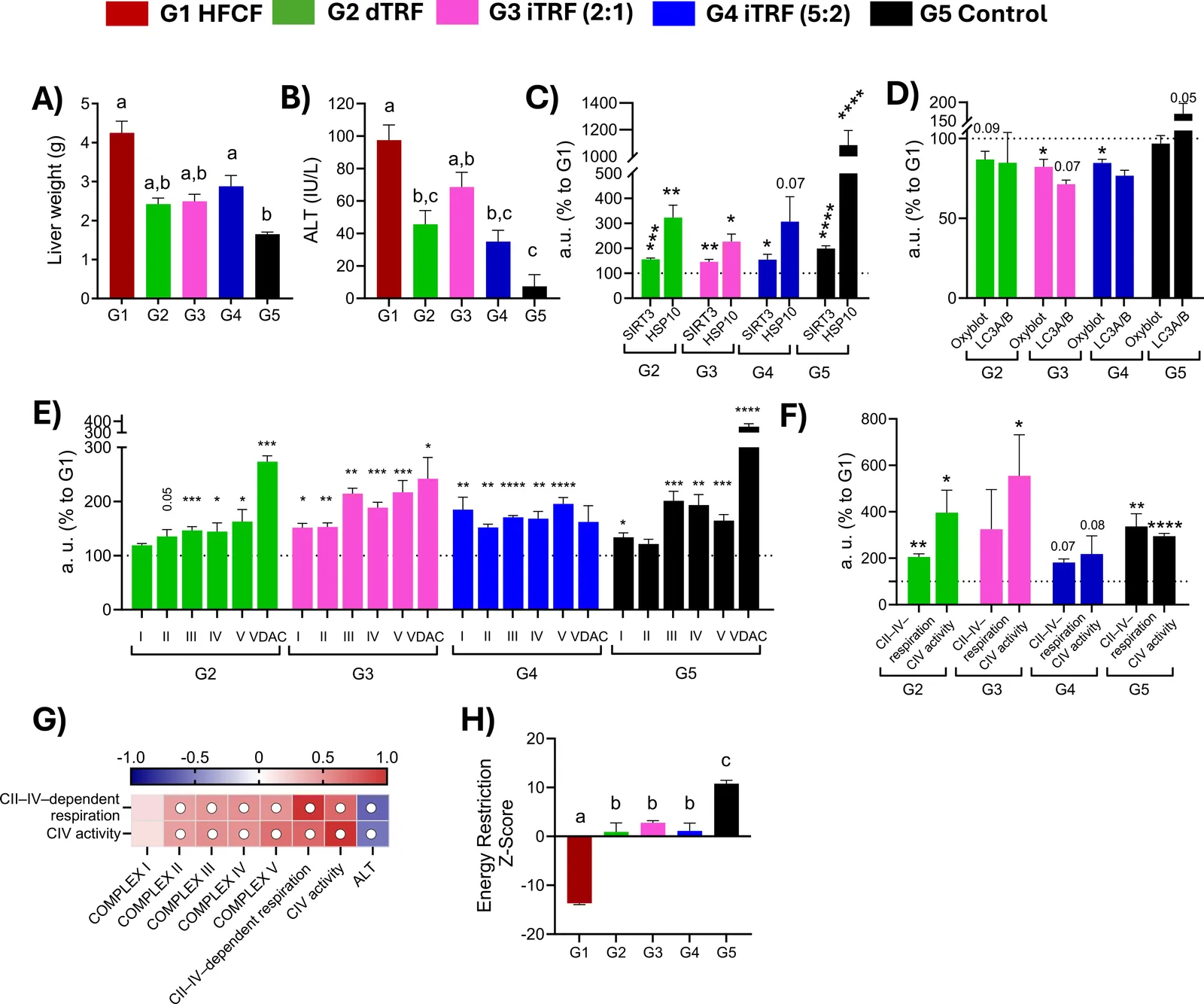
**- Both iTRF and dTRF promoted liver physiology remodeling towards SD control** mice**. A)** Liver weight. **B)** Plasma ALT levels. **C-D)** SIRT3 and HSP10 (C) and Oxyblot and LC3A/B (D) protein quantification by Western Blot. **E)** Mitochondrial respiratory complexes (I, II, III, IV and V) and VDAC protein quantification by Western Blot. **F)** Macro-complex II-III-IV and complex IV maximum respiration (accessed by SeaHorse) of all intervention groups comparing with G1 set at 100% (dotted line). **G)** Complex II-III-IV and complex IV dependent respiration correlations with individual mitochondrial respiratory complexes and plasma ALT. **H)** Energy Restriction z-score calculated by the sum of z-scores of the different parameters represented on Figure 3 (liver weight, plasma ALT, Western Blot quantification of mitochondrial respiratory complexes (I, II, III, IV and V), VDAC, SIRT3, HSP10, LC3A/B and Oxyblot, and complex II-III-IV and complex IV dependent respiration. Data are presented as the mean ± SEM (A-F, and H) or Pearson correlation value heatmap with white dot indicating statistical difference (G). One-way ANOVA tests followed by Tukey's multiple comparation test were implemented to compare data from panel H, or Kruskal–Wallis's test followed by Dunn's multiple comparation test for panels A and B if one or more groups did not follow normal distribution assessed by Shapiro–Wilk test. On panel C, E and F a t-test or Mann Whitney test (if one or more groups did not follow normal distribution) comparing each group with HFCF group (set at 100%, dotted line). On A, B and H, columns that do not share a common letter (a, b, c) are statistically different (p < 0.05). On C, D, E and F, asterisks indicate significant difference (∗p < 0.05; ∗∗p < 0.01; ∗∗∗p < 0.001; ∗∗∗∗p < 0.0001) and if p-value is between 0.05 and 0.1 the p-value is indicated above the column.

As expected, SD mice displayed reduced liver weight vs. HFCF mice (61% reduction, Figure 3A). To a lesser extent, all TRF interventions led to a reduction in hepatic weight, with decreases of 43%, 41%, and 32% observed for dTRF, iTRF 2:1 and iTRF 5:2 groups, respectively (Figure 3A). Levels of alanine aminotransferase (ALT), an indicator of liver function, were also found lower in SD mice and TRF groups vs. HFCF mice (Figure 3B). When compared to HFCF mice, hepatic protein levels of SIRT3 (a fasting-associated marker) and HSP10 (a reported SIRT3 substrate [50], were found elevated across all TRF interventions (Figure 3C and Suppl. Fig. 5A). A favorable molecular profile in all TRF groups was also suggested by attenuated oxidative stress and a possible autophagy induction, as determined by reduced carbonylated proteins and a trend but not significant reduction of LC3A/B (Figure 3D and Suppl. Fig. 5B). Hepatic protein levels of several markers for mitochondrial respiratory complexes (NDUFB8, SDHB, UQCRC2, MTCO1, and ATP5A from Complexes I to V, respectively) as well as of VDAC, a marker of mitochondrial mass, were found elevated in SD mice and also across all TRF interventions (Figure 3E and Suppl. Fig. 5C–D). Thereafter, hepatic mitochondria were isolated to assess oxygen consumption rates using the gold-standard Seahorse assay. Respiration involving complexes II + III + IV in response to succinate plus rotenone, as well as complex IV-linked maximal respiration in response to N,N,N′,N′-tetramethyl-p-phenylenediamine (TMPD) plus ascorbic acid were significantly elevated in SD mice and in dTRF mice vs. HFCF mice (Figure 3F and Suppl. Fig. 5F). Exposure to intermittent TRF schedules led to consistent trends, reaching statistical significance for CIV in iTRF 2:1 mice (Figure 3F). Notably, oxygen consumption rates for both CII + III + IV and CIV were positively associated with protein levels of mitochondrial respiratory complexes and inversely with circulating ALT levels (Figure 3G). Likewise, ALT levels showed a positive association with liver weight (Suppl. Fig. 5H). Finally, a collective Z-score was calculated representing the molecular signature of energy restriction. As shown in Figure 3H, all TRF regimes led to a significant elevation of this score vs. HFCF mice, with no significant differences detected between TRF regimes, (Figure 3H).

**Figure 4:**
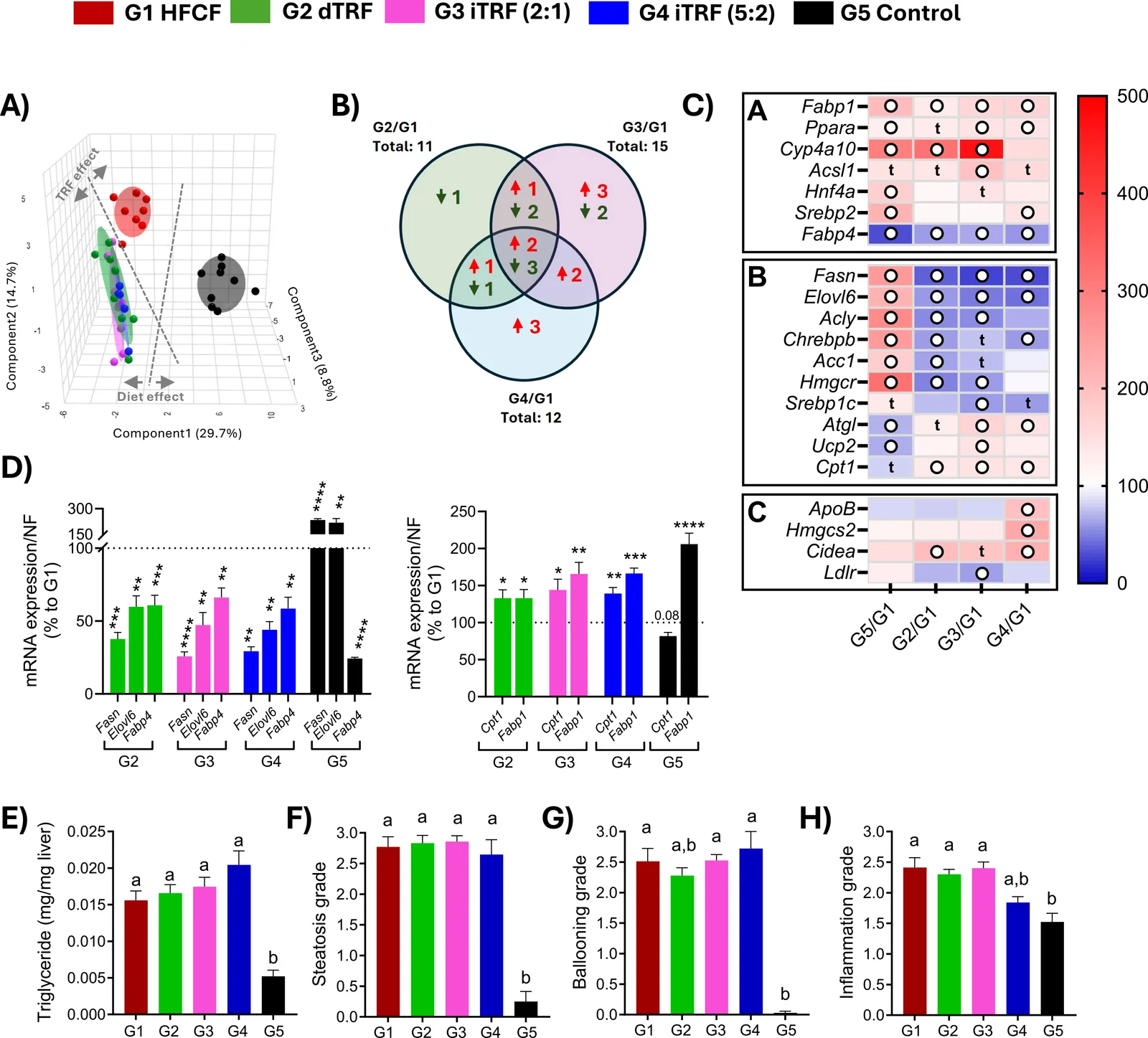
**- iTRF interventions and dTRF promoted transcriptional shifts toward lipid utilization in liver. A)** Partial Least Squares Discriminant Analysis (PLS-DA) of the 43 genes measured associated with lipid metabolism. **B)** Venn diagram representing the significantly altered genes from liver samples from TRF groups (dTRF, iTRF 2:1 and iTRF 5:2) compared to HFCF group. **C)** Heatmap clustering of the 21 differently expressed genes between at least one TRF condition and HFCF group (set at 100%), indicating the number of genes upregulated in red and downregulated in green. **D)** Differently expressed genes across all TRF conditions compared to HFCF group (set at 100%). **E)** Liver triglycerides content. **F–H)** Histological evaluation of liver steatoses grade (F), ballooning (G), and inflammation (H). Data are presented as PLS-DA components score (A), as heatmap of the means (C) or mean +SEM (D–F). One-way ANOVA tests followed by Tukey's multiple comparation test were implemented to compare data from panels E and H), or Kruskal–Wallis's test followed by Dunn's multiple comparation test for panels F and G if one or more groups did not follow normal distribution assessed by Shapiro–Wilk test. On panel C and D, a t-test or Mann Whitney test (if one or more groups did not follow normal distribution) comparing each group with HFCF group (set at 100%). On panel C, statistical significance was considered when p-value <0.05 and represented as white circles, while p < 0.1 was indicated as a trend (t). On panel D, asterisks indicate significant difference vs. HFCF group (∗p < 0.05; ∗∗p < 0.01; ∗∗∗p < 0.001; ∗∗∗∗p < 0.0001) and if p-value is between 0.05 and 0.1 the p-value is indicated above the column. On panels E, F, G and H, columns that do not share a common letter (a, b, c) are statistically different (p < 0.05).

Next, given that TRF-fed mice showed greater reliance on lipid metabolism, the expression of 43 key genes participating in the metabolism of lipid were quantified using the Standard BioTools platform (Supplementary Table 5). As shown in Figure 4A, Partial Least Squares Discriminant Analysis (PLS-DA) of the resulting gene expression matrix showed two main components, likely reflecting (i) the effect of diet (HFCF vs. SD; Component 1, 29,7%) and (ii) the impact of TRF interventions (Component 2, 14,7%; in which dTRF, iTRF 2:1 and iTRF 5:2 interventions overlap). Out of 43 genes, 53% of them (23 genes) were differentially expressed in SD mice compared to HFCF mice (visualized in the volcano plot, Suppl. Fig. 6A; 17 upregulated and 6 downregulated). Genes contributing to the effects of TRF (defined as those significantly modulated in at least one TRF condition compared to HFCF) were represented using a Venn diagram (Figure 4B) and further visualized in a heatmap (Figure 4C), uncovering the existence of 21 genes classified in two major gene clusters: genes whose expression patterns resembled those in SD-mice (Cluster A, Figure 4C) and genes with opposite regulation to SD-mice (Cluster B, Figure 4C). A third, residual gene set was identified in which few genes, including *Cidea*, were specifically modulated by TRF interventions vs. HFCF, independently of SD effects (Cluster C, Figure 4C). Among these clusters, daily TRF regulated 11 DEGs, with 5 of them (45%) consistently regulated across all TRF regimens (Figure 4D*; Fabp1* and *Fabp4* from Cluster A; *Fasn, Elovl6,* and *Cpt1* from Cluster B). Overall, the majority of the 21 identified genes displayed a consistent pattern of expression across all TRF interventions, with changes generally occurring in the same direction and within a similar range of magnitude regardless of the specific TRF regimen (Figure 4A,B). Altogether, our findings demonstrate that both daily and intermittent TRF promoted ER-related molecular signatures and transcriptional shifts toward lipid utilization, ultimately improving liver function as evidenced by reduced ALT. Despite these advantages, hepatic triglyceride levels remained unexpectedly unchanged in both HFCF and TRF-treated mice, in sharp contrast with a marked reduction found in SD mice (Figure 4E). Histological quantification of steatosis, hepatocellular ballooning and inflammatory grade revealed similar patterns: while SD mice showed marked improvements, these parameters remained unchanged in mice subjected to any of the TRF interventions (Figure 4F–H and Suppl. Fig. 6B).

**Figure 5:**
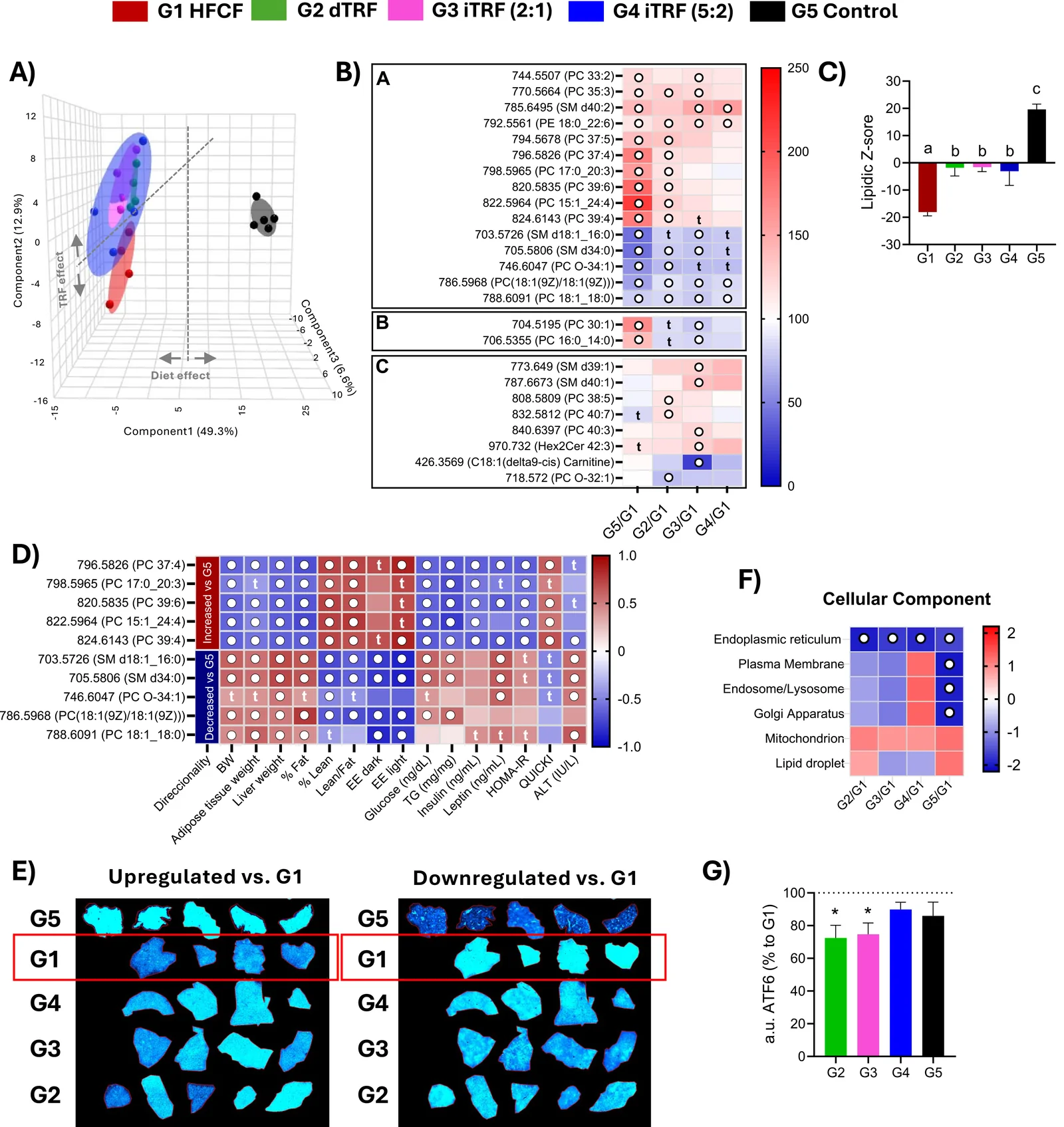
**- iTRF interventions and dTRF promoted similar lipid profile modulation in liver. A)** Partial Least Squares Discriminant Analysis (PLS-DA) of the 291 identified lipids **B)** Heatmap of the 25 lipids significantly altered in at least one TRF condition vs HFCF (set at 100%), grouped in clusters according to its directionality. **C)** Lipid z-score calculated by the sum of z-scores of the 25 lipides defining clusters A, B and C from panel B. **D)** Correlations of a subset of 5 upregulated and 5 downregulated lipids, compared to HFCF, with relevant metabolic parameters. **E)** Mass spectrometry imaging (MSI) visualization of differential lipid abundance in liver tissue sections. (Left) Spatial mapping of hepatic lipid species significantly upregulated relative to the control group (G1). (Right) Spatial mapping of hepatic lipid species significantly downregulated relative to G1. Rows represent individual experimental, with multiple liver lobe replicates shown per row. The color intensity scale reflects relative lipid ion abundance, where bright cyan denotes maximum structural lipid intensity/accumulation and dark blue/black denotes minimal detection or suppression. **F)** Lipids subcellular distribution assessed using LipidSig 2.0 database **G)** Liver Western Blot protein quantification of ATF6 (% to HFCF). Data are presented as PLS-DA components score (A), as heatmap of the means (B), mean ± SEM (C, G), Pearson correlation value heatmap (E) or heatmap of cellular components lipid composition enrichment score (F). One-way ANOVA tests followed by Tukey's multiple comparation test were implemented to compare data from panel C. On panel A and G, a t-test or Mann Whitney test (if one or more groups did not follow normal distribution) comparing each group with HFHC group set at 100%. On panel B, E and F, statistical significance was considered and represented as white circles when p-value <0.05, while p < 0.1 was indicated as a trend (t). On panel C, columns that do not share a common letter (a, b, c) are statistically different (p < 0.05). On panel G, asterisk indicates significant difference (∗p < 0.05).

To further investigate these observations, we next assessed comprehensive lipidomic profiling using MS-Imaging, which allowed us to detect 292 lipidic variants, with 100 of them successfully annotated using reference databases (Suppl. Table 6). As shown in Figure 5A, PLS-DA analysis of the resulting lipid intensity matrix showed a pattern consistent with that observed in the transcriptomic analysis (Figure 3F), identifying two main components: (i) Component 1 (49.3%), likely reflecting the effect of diet (HFCF vs. SD); and (ii) Component 2 (12,9%), likely reflecting the influence of TRF interventions. Out of 292 lipids analyzed 69% were differentially expressed in SD mice compared to HFCF mice (visualized in the volcano plot, Suppl. Fig. 6C; 136 upregulated and 65 downregulated). In addition, we identified 25 annotated lipids contributing to the effects of TRF (i.e., those significantly modulated in at least one TRF group compared to HFCF mice). These lipids segregated into the same well-defined clusters (Figure 5B; Clusters A, B and C), with most showing similar levels and equal directionality of modulation regardless of the specific TRF schedule (Figure 5B)]. Indeed, evaluation of a lipidic Z-score comprising these 25 lipids indicated a significant elevation across all TRF regimens vs. HFCF mice, with no significant differences detected between TRF regimes (Figure 5C). In addition, we found that a subset of these lipids (total of 10, 5 upregulated and 5 downregulated) were associated with multiple metabolic parameters (including body weight, body composition, and circulating biomarkers related to metabolic health, among others) (Figure 5D). Visualization in liver sections of lipid abundance from this “core” signature is shown in Figure 5E. The left and right panels display the summed normalized abundance of the upregulated and downregulated differentially expressed lipids from this “core”, respectively.

Next, we evaluated the subcellular distribution of specific lipids using the LipidSig 2.0 database. Interestingly, returning to SD diet was associated with a broad redistribution of lipids across multiple cellular compartments, including the endoplasmic reticulum, plasma membrane, endosomes/lysosomes, and the Golgi apparatus (Figure 5F). In contrast, TRF interventions produced more localized effects, with significant changes detected predominantly in lipid distribution within the endoplasmic reticulum (Figure 5F). Interestingly, endoplasmic reticulum-associated lipids showed interrelated patterns, consistent with a degree of coordination within this lipid network (Suppl. Fig. 6D). In addition, hepatic protein levels of ATF6, a well-known stress marker of the endoplasmic reticulum, were found reduced across TRF interventions vs. HFCF- mice (Figure 5G and Suppl. Fig. 6E).

## 4 Discussion

TRF is increasingly contemplated as a clinically relevant strategy for preventing and treating obesity and certain metabolic disorders, including MASLD [38,51,52]. Among other benefits, TRF promotes multi-systemic and multi-organ adaptations leading to improved weight control, glucose and lipid handling, as well as restoration of whole-body metabolic homeostasis [53,54]. The use of intermittent cycles and/or very low-calorie cycling protocols have been extensively studied as energy restriction-based strategies [23,24,55,56]. In the specific context of TRF, several studies have compared differential effectiveness between early, mid, and late (and/or self-selected) TRF [[57], [58], [59], [60]], but the implementation of more pragmatic, appealing, and readily achievable intermittent TRF schedules remains rather unexplored [[30], [31], [32]]. Herein, we implemented two intermittent TRF schedules (2:1 and 5:2 cycles) to provide the first head-to-head evidence that iTRF recapitulates key metabolic and physiological benefits of daily TRF in mice with pre-existing metabolic dysfunction promoted by prolonged high-fat, high-cholesterol, high-fructose (HFCF) feeding prior to the initiation of TRF interventions.

From a physiological perspective, our results corroborate that, after 25 weeks of HFCF, dietary normalization to a standard diet represents the superior intervention, leading to a prominent improvement across the measured parameters. To a lesser extent, - and independent of TRF scheduling – we demonstrate that both daily and intermittent TRF produced significant improvements in body weight, body composition, glucose homeostasis, and physical performance, a pattern that is recapitulated by the metabolic z-score (Figure 2H), altogether indicating a global enhancement of metabolic and systemic health [31]. Of note, all TRF variants tested in our study are associated with reduced food intake. Conceptually, classical studies have demonstrated that daily TRF protects from diet-induced obesity and metabolic dysfunction without a net reduction in total caloric intake, suggesting that prolonged daily fasting periods rather than the total amount of calories consumed represent the critical factor responsible for the enhancement of metabolic health [38,61]. This is also documented for other energy restriction-based strategies [62,63]. However, subsequent work discloses more heterogeneous interpretation, in which the impact of energy restriction on health and lifespan is influenced by spontaneous changes in food intake and other multiple variables including gender, age-onset, diet, genotype and regimen imposed [32,49,64,65]. For example, Chaix and colleagues found that TRF can change how much and how quickly animals eat, meaning that TRF-mediated benefits may partially arise from reduced food intake rather than the eating timing [32,66]. This complexity is also evident in human trials, where in some studies suggest that reductions in energy intake account for a large proportion of the benefits attributed of TRF [67,68]. In other trials, the absence of data quantifying energy intake limits the ability to determine whether TRF benefits result from the fasting period itself, reduced energy intake, their combination, and/or other factors [29,31,69,70]. In our study, the convergence of outcomes between daily and intermittent TRF regimens may be partially explained by comparable reductions in energy intake between TRF variants. However, due to the absence of a pair-fed control group, we cannot fully disentangle the contribution of caloric restriction from that of feeding timing, and therefore the extent to which the observed benefits are driven by TRF per se should be interpreted with caution. Nonetheless, several physiological improvements persisted after normalization to cumulative calorie intake, suggesting that, beyond caloric intake alone, the feeding schedule itself contributes to the observed benefits. It has also been documented that metabolic benefits of TRF may persist even if TRF is not applied every day, suggesting a degree of metabolic resilience or “fasting memory” in the system [32]. This is also reported for other energy restriction-based interventions, which argue that adaptive changes at molecular and physiological states - in metabolism, inflammation, and circadian regulation - can persist beyond the fasting window, and sometimes beyond the intervention period [62,63,66,71,72]. In this scenario, comparable physiological outcomes observed between daily and intermittent TRF may be also partially explained by the existence of a TRF-induced “metabolic memory”, although further studies are required to validate this hypothesis. Finally, a metabolic “ceiling effect” limiting weight loss has been consistently described in previous preclinical [32] and clinical reports [54,67,73] in which energy restriction is implemented. This effect was also observed in our study for daily and intermittent TRF, which may also account for the similar physiological outcomes found between both regimens.

TRF recapitulates the biological hallmarks of energy restriction [24,29,70,74]. These include metabolic switching, activation of nutrient-sensing pathways, mitochondrial remodeling, circadian alignment, resistance to cellular stress, epigenetic remodeling and improved proteostasis, altogether contributing to long-term metabolic resilience [24,66]. Consistent with previous literature, daily TRF markedly activated several hallmarks of ER in our study. Activation of these hallmarks was also observed when intermittent TRF protocols were implemented, a pattern that is recapitulated by the energy restriction z-score (Figure 3H). It should be stated that, to minimize circadian variability and to maximize comparability across dietary groups, molecular analyses were performed at a single time point, immediately before the initiation of the dark phase, when TRF mice had completed 16 h of fasting, and SD and HFCF mice had completed 14 h of light/inactive phase, a period of low spontaneous feeding activity. At this point, the expression of hepatic SIRT3 and HSP10 were found elevated [50,75]. SIRT3 regulates hepatic mitochondrial fatty acid β-oxidation [76,77] and represents the major mitochondrial sirtuin responsible for the protection from stress-induced mitochondrial activity and energy metabolism [78]. By preserving SIRT3 signaling, mitochondrial HSP60 promotes fatty acid oxidation and represses mitochondrial stress and inflammation [75]. In this context, the concurrent increase in SIRT3 and HSP60 observed here is consistent with improved mitochondrial function and lipid handling, mechanisms that may contribute to protection against MASLD development, [50,79], although, in the absence of functional validation, these findings should be interpreted with caution. Consistently, our data demonstrate reduced protein oxidation, increased expression of VDAC and markers of mitochondrial respiratory complexes, together with enhanced mitochondrial activity (specifically via macrocomplex II + III + IV and complex IV), reflecting improved mitochondrial abundance and efficiency [80,81]. In line with this notion, the organization of complexes III and IV into respiratory supercomplexes has been linked to respiratory flux, fatty acid oxidation-supported electron transfer, and mitochondrial redox homeostasis [[82], [83], [84]]. In parallel, TRF induced a transcriptional profile that separated from both HFCF and SD *ad libitum* feedings, highlighting the emergence of a third, distinct metabolic state shared by all TRF variants. *Pparα* and *Fabp1* expression were found elevated, whereas expression of *Fabp4* was reduced. By activating *Pparα*, increased *Fabp1* expression during fasting is reported to ensure the high influx of fatty acids is promptly channeled towards energy production (oxidation), thereby preventing their harmful accumulation or lipotoxicity [85,86]. Likewise, reduced *Fabp4* has been shown to associate with reduced hepatic metabolic and inflammatory stress and with restored mitochondrial function [87,88]. On the other hand, *Fasn, Acly,* and *Elovl6* expression*,* were found increased in SD vs. HFCF mice, consistent with a reactivation of lipogenesis in SD mice once the lipotoxic and inflammatory stress induced by HFCF feeding was removed [89,90], which represents an apparent paradox well recognized in metabolic research [32,[91], [92], [93], [94], [95]]. In contrast, *Fasn, Acly* and *Elovl6* were markedly downregulated in response to TRF schedules, suggesting that TRF attenuates *de novo* lipogenesis and elongation of very long chain fatty acids, [66,96]. We also detected an increase in *Cidea* expression, which could represent a TRF-adaptive mechanism to fine-tune lipid handling during fasting [97]. Collectively, these observations are compatible with a shift from lipid synthesis toward increased mitochondrial oxidative pathways, although further functional studies are required to confirm this interpretation.

Of note, mitochondrial respiration is shown to inversely correlate with circulating ALT2 pathways, which together with the mitochondrial pyruvate carrier (MPC), may act as complementary routes to sustain anaplerosis under conditions of nutrient scarcity. In line with this notion, we observed an association between improved respiration and lower ALT levels, suggesting a link between ALT activity, mitochondrial performance, and reduced hepatocellular stress [[98], [99], [100], [101]].

It should be discussed that, despite overall TRF-mediated benefits across multiple layers (physiological, metabolic, and molecular), hepatic lipid accumulation, hepatocellular ballooning, and hepatic inflammation, all of them contributing to a higher histological inflammatory grade [102], were found unexpectedly unchanged between continuous HFCF feeding and TRF interventions. This observation highlights an important limitation of the present study, suggesting that TRF alone may not be sufficient to reverse established or advanced stages of MASLD. While several preclinical and clinical studies have documented tissue-level recovery under TRF conditions [55,[103], [104], [105], [106], [107]] and improvements in triglyceride metabolism [[108], [109], [110], [111], [112]], some TRF studies report no improvement - or even a deterioration - in lipid metabolism, particularly reflected by adverse changes in serum triglyceride levels [23,54,57,[113], [114], [115], [116], [117], [118]]. For example, limited TRF effects were found on histological improvement in hepatic architecture in a choline-deficient HFD-induced mouse model of MASH (Metabolic Dysfunction-Associated Steatohepatitis) [119]. In patients diagnosed with MAFLD (Metabolic Dysfunction-Associated Fatty Liver Disease) by abdominal ultrasound, 12 weeks of TRF did not affect liver stiffness or serum levels of cholesterol, HDL and LDL, despite body weight, fat mass and serum TG were found reduced [108]. In women and men with overweight and obesity, TRF had no effect secondary outcomes such as cholesterol, HDL, LDL, and various circulating biomarkers related to metabolic health (glucose, insulin, Hba1c and the HOMA-IR index), despite body weight was also found reduced [120]. In a different trial, early TRF increased plasma triglycerides, and had no effect on arterial stiffness, LDL and HDL cholesterol levels, or markers of inflammation, [57]. Elevated plasma TG were also reported in a mouse model of dietary-induced obesity exposed to TRF [121]. When viewed together, it appears that TRF effects on dyslipidemia and histological benefits are context-dependent and vary with dietary model, disease stage and intervention length [122,123]. Nonetheless, continuous development of high throughput techniques offers more precise approaches to assessing lipidomic remodeling reflecting improved lipid handling and restored lipid homeostasis [57,121,124]. Herein, the hepatic lipidome of the various TRF schedules segregated distinctly from that of animals maintained on continuous HFCF feeding, as well as from those fed a SD. This pattern is in concordance with our transcriptomic data and supports a third, distinct metabolic state indicative of favorable metabolic adaptations, also in line with previous reports [121]. Notably, lipidomic profiles from all TRF regimens clustered together, indicating a shared remodeling signature that is captured by the lipidomic z-score (Figure 5C). Furthermore, TRF appears to induce a redistribution of lipid species associated with the endoplasmic reticulum. Whereas chronic overnutrition promotes endoplasmic reticulum stress in hepatocytes [38,125,126], fasting enhances the interactions of endoplasmic reticulum with other organelles, including mitochondria and peroxisomes, to support cellular homeostasis and metabolic health [127]. In addition, several saturated and monounsaturated phosphatidylcholine (PC) species - including PC (18:1_18:0), PC (18:1 (9Z)/18:1 (9Z)) and the choline plasmalogen PC O-34:1, were found reduced following TRF interventions. These changes are compatible with shifts in membrane lipid saturation. In line with this notion, the saturation status of membrane lipids is known to influence membrane biophysical properties and to attenuate chronic oxidative and endoplasmic reticulum stress, partly via downregulation of unfolded protein response (UPR) signaling [127,128]. Concomitantly, we found reduced expression of the endoplasmic reticulum stress sensor ATF6 in response to TRF interventions. ATF6 is typically reported to be upregulated during endoplasmic reticulum stress in MASLD models [129,130] and implicated in pathological liver remodeling [131]. TRF interventions also induced a significant reduction in key sphingomyelin (SM) species [SM(d18:1/16:0) and SM(d34:0)], lipid classes closely linked to ceramide metabolism, hepatic lipotoxicity and insulin resistance [132,133]. Reduction of these SM species are associated with limited sphingolipid accumulation, the latter closely associated with metabolic dysfunction [[134], [135], [136]]. In parallel, TRF schedules increased polyunsaturated lipid species [PC (39:4), PE (18:0_22:6), and PC (35:3)]. Incorporation of polyunsaturated fatty acids are shown to favor membrane remodeling, fluidity, and resistance to oxidative stress [137]. Notably, TRF interventions increased the levels of the Docosahexaenoic Acid (DHA)-containing lipid PE (18:0_22:6), which is reported to be enriched in mitochondrial membranes, has been implicated in maintaining mitochondrial integrity and endoplasmic reticulum-mitochondria communication through mitochondria-associated membranes (MAMs), and has been associated to mitochondrial function and anti-inflammatory capacity [[138], [139], [140]].

In sum, our results demonstrate that iTRF recapitulates the benefits of dTRF. Both iTRF and dTRF activated several hallmarks of energy restriction and promoted coordinated transcriptional and lipidomic remodeling to enhance hepatic mitochondrial function, metabolic flexibility, and systemic health. Captured from independent data layers, we generated three different composite scores reflecting physiological/metabolic status, energy restriction, and lipidomic remodeling. Interestingly, composite scores showed consistent associations between them, indicating a unified TRF metabolic state tightly interconnected across physiological and biological scales. Finally, we identified a subset of lipids associated with metabolic parameters and systemic health. Monitoring this lipid signature may provide useful information during disease progression or to assess treatment efficacy.

## 5 Study limitations

This study was conducted exclusively in male C57BL/6 J mice, as females are relatively protected against obesity and insulin resistance; however, future studies should assess TRF responses in female models to account for potential sex-specific metabolic effects. TRF-mediated improvement in physical performance might be partially explained by changes in body weight and adiposity, with additional analyses needed to determine intrinsic improvements in muscle or neuromuscular function. The inclusion of a pair-fed control group would help to further dissect the relative contribution of caloric intake vs. feeding schedule itself. In addition, molecular analyses were performed at a single time point, immediately before the initiation of the dark phase, when TRF mice had completed 16 h of fasting, and SD and HFCF mice had completed 14 h of light/inactive phase (Supplemental Fig. 1A). This design limits the assessment of circadian variation and precludes evaluation of postprandial responses and therefore may not fully capture TRF-induced molecular dynamics across the daily cycle. Intermittent TRF regimens with a lower cumulative TRF exposure than 20 days per month were not evaluated and may yield distinct metabolic outcomes. Likewise, mitochondrial isolation from frozen tissue may affect functional integrity, although it still allows for meaningful comparative analyses. Finally, lipidomic profiling was limited to anionic lipid species due to technical limitations inherent to the analytical approach, precluding a comprehensive analysis of the entire hepatic lipidome.

## CRediT authorship contribution statement

**Andre Sarmento-Cabral:** Writing – review & editing, Methodology, Formal analysis, Data curation. **Marta G. Novelle:** Writing – review & editing, Writing – original draft, Investigation, Formal analysis. **Luz Marina Sánchez-Mendoza:** Formal analysis, Data curation. **Natalia Herman-Sanchez:** Formal analysis, Data curation. **Samanta Lozano de la Haba:** Formal analysis, Data curation. **Marta Rodriguez-Vazquez:** Formal analysis, Data curation. **Pablo Iturbe:** Formal analysis, Data curation. **Eduardo Chicano-Gálvez:** Formal analysis, Data curation. **Francisco Javier Cubero:** Formal analysis, Data curation. **Raul M. Luque:** Writing – review & editing, Formal analysis. **Jose Cordoba-Chacon:** Writing – review & editing, Formal analysis. **Juan L. Lopez-Canovas:** Writing – review & editing, Formal analysis. **Jose M. Villalba:** Writing – review & editing, Formal analysis. **Manuel D. Gahete:** Writing – review & editing, Supervision, Resources, Conceptualization. **Alberto Diaz-Ruiz:** Writing – review & editing, Writing – original draft, Supervision, Resources, Conceptualization.

## Ethics declaration

This study was conducted in accordance with the ARRIVE (Animal Research: Reporting of In Vivo Experiments) guidelines. This study was approved by the University of Cordoba and the Regional Government Research Ethics Committees. (Approval No. 15/05/2018/088 and 08/03/2022/033).

## Declaration of competing interest

The authors declare that they have no known competing financial interests or personal relationships that could have appeared to influence the work reported in this paper.

## Data Availability

Data will be made available on request.
