# Supplementary material for: Intermittent time-restricted feeding recapitulates the physiological benefits of daily time-restricted feeding in male mice with chronic metabolic disease
Source: Mol Metab. 2026 Aug 25;113:102432. doi: 10.1016/j.molmet.2026.102432 (PMC13587810; doi:10.1016/j.molmet.2026.102432)

Supplemental Figure 1, related to Figure 1

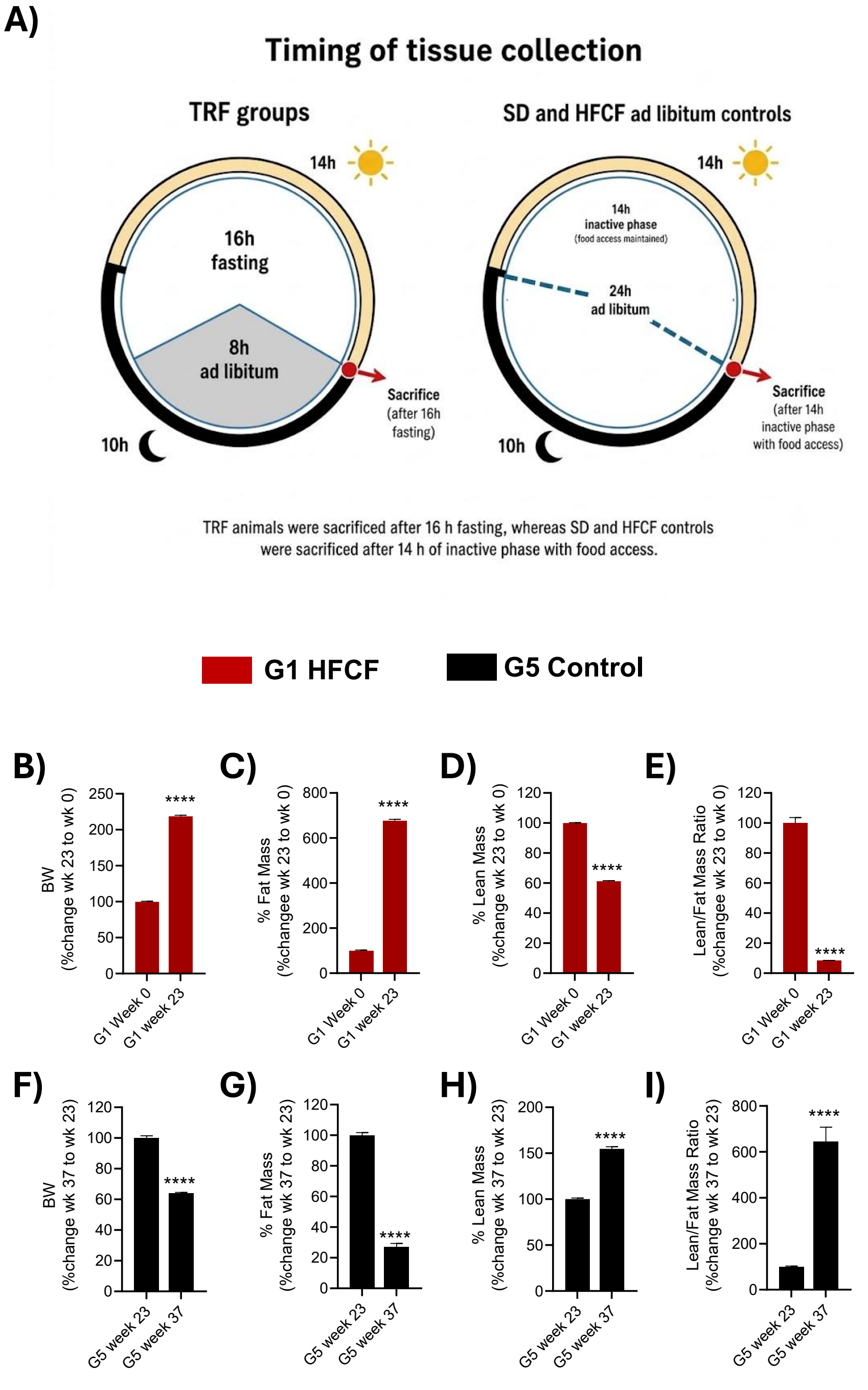

Supplemental Figure 2, related to Figure 1

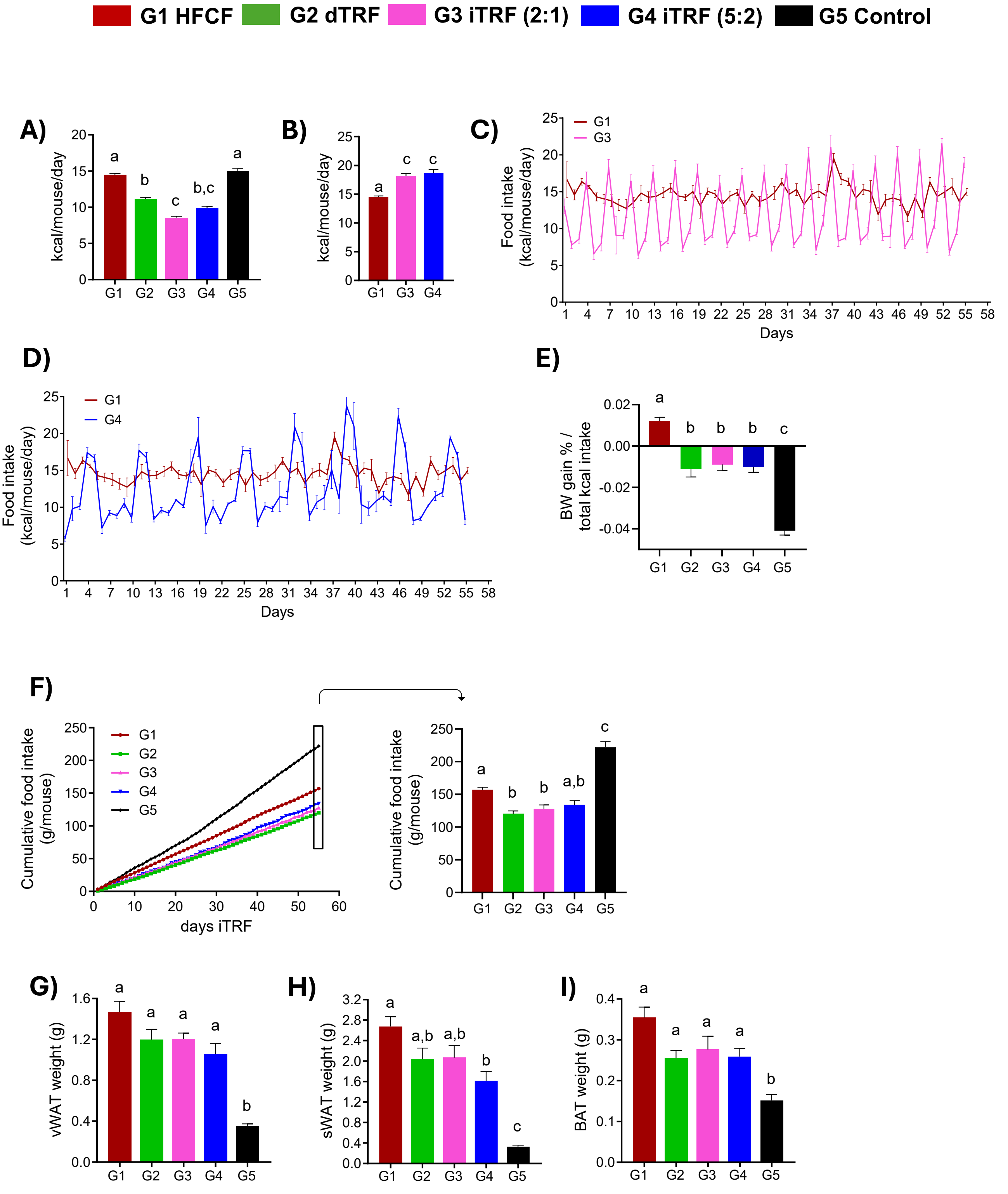

Supplemental Figure 3, related to Figure 1

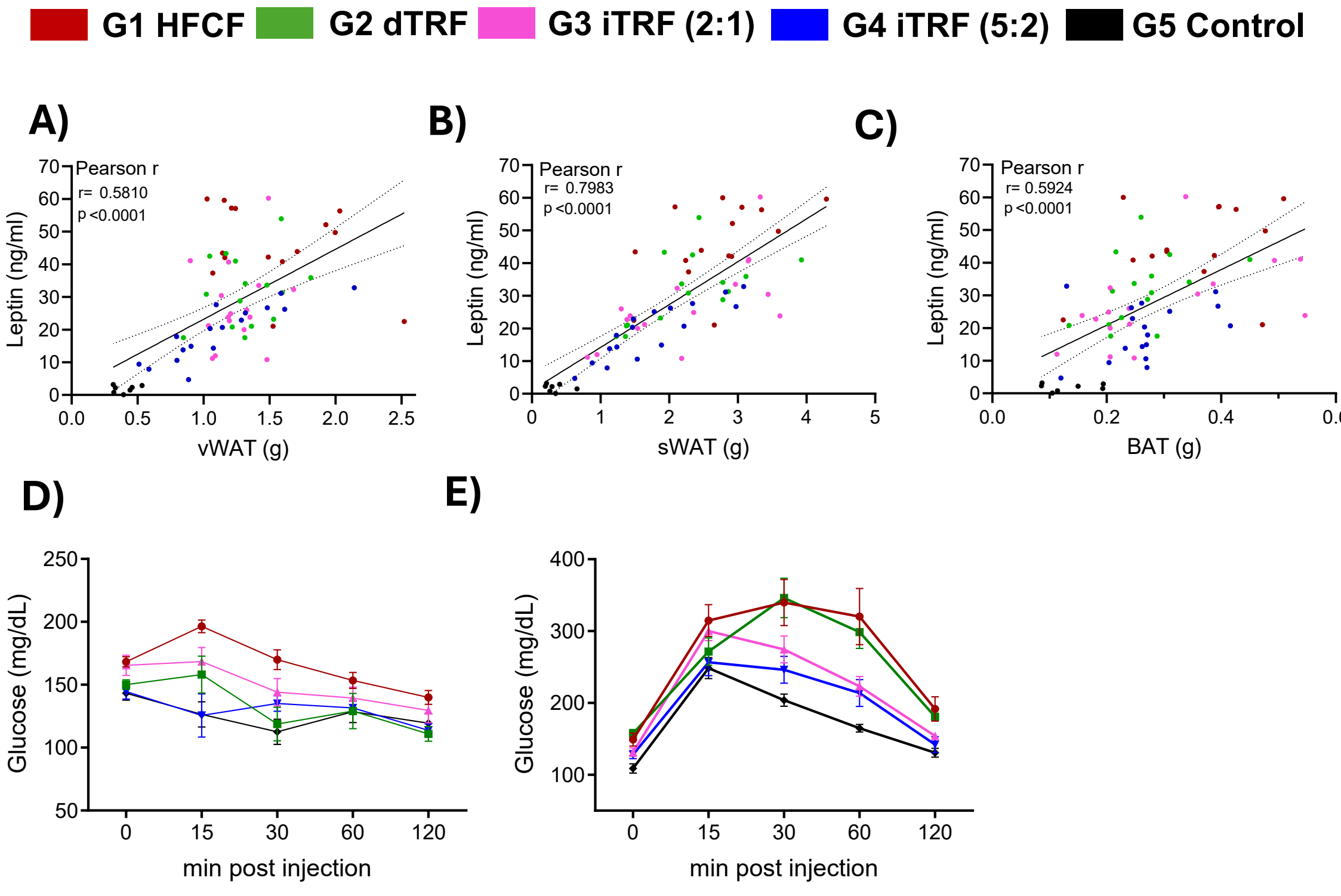

Supplemental Figure 4, related to Figure 2

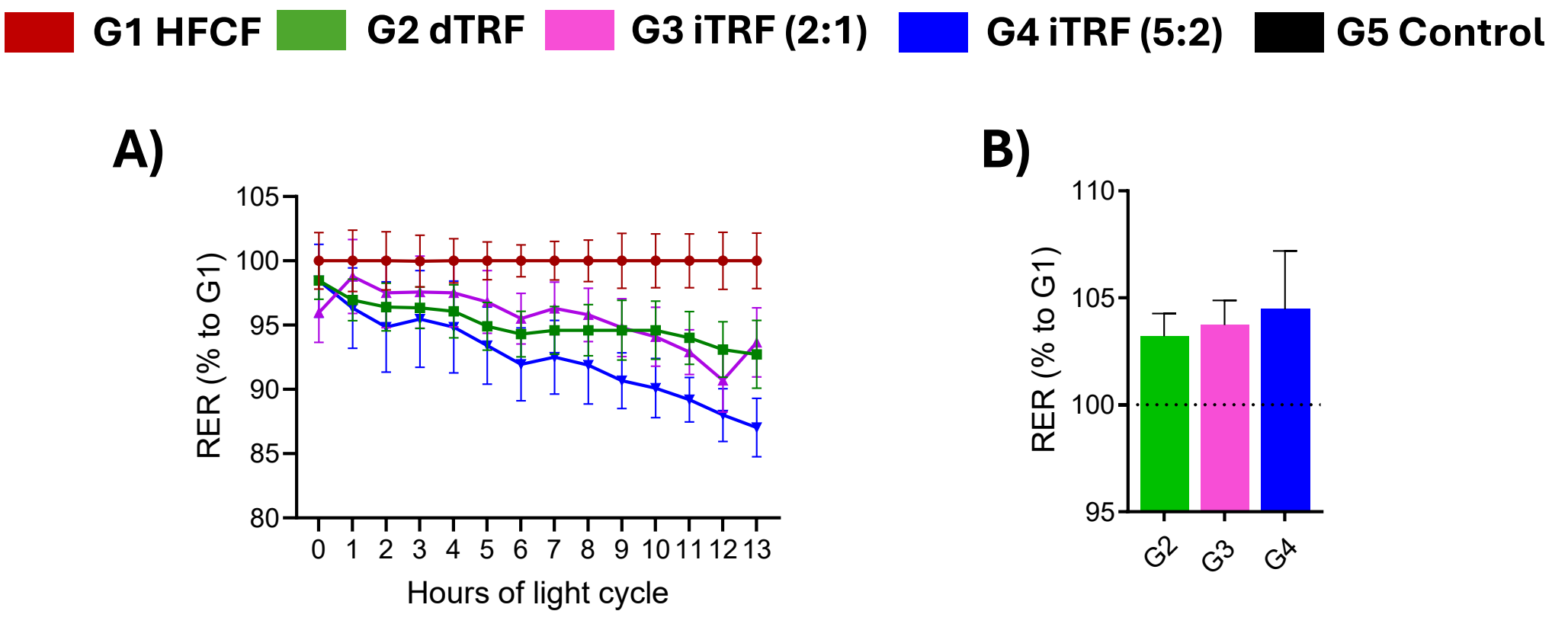

Supplemental Figure 5, related to Figure 2

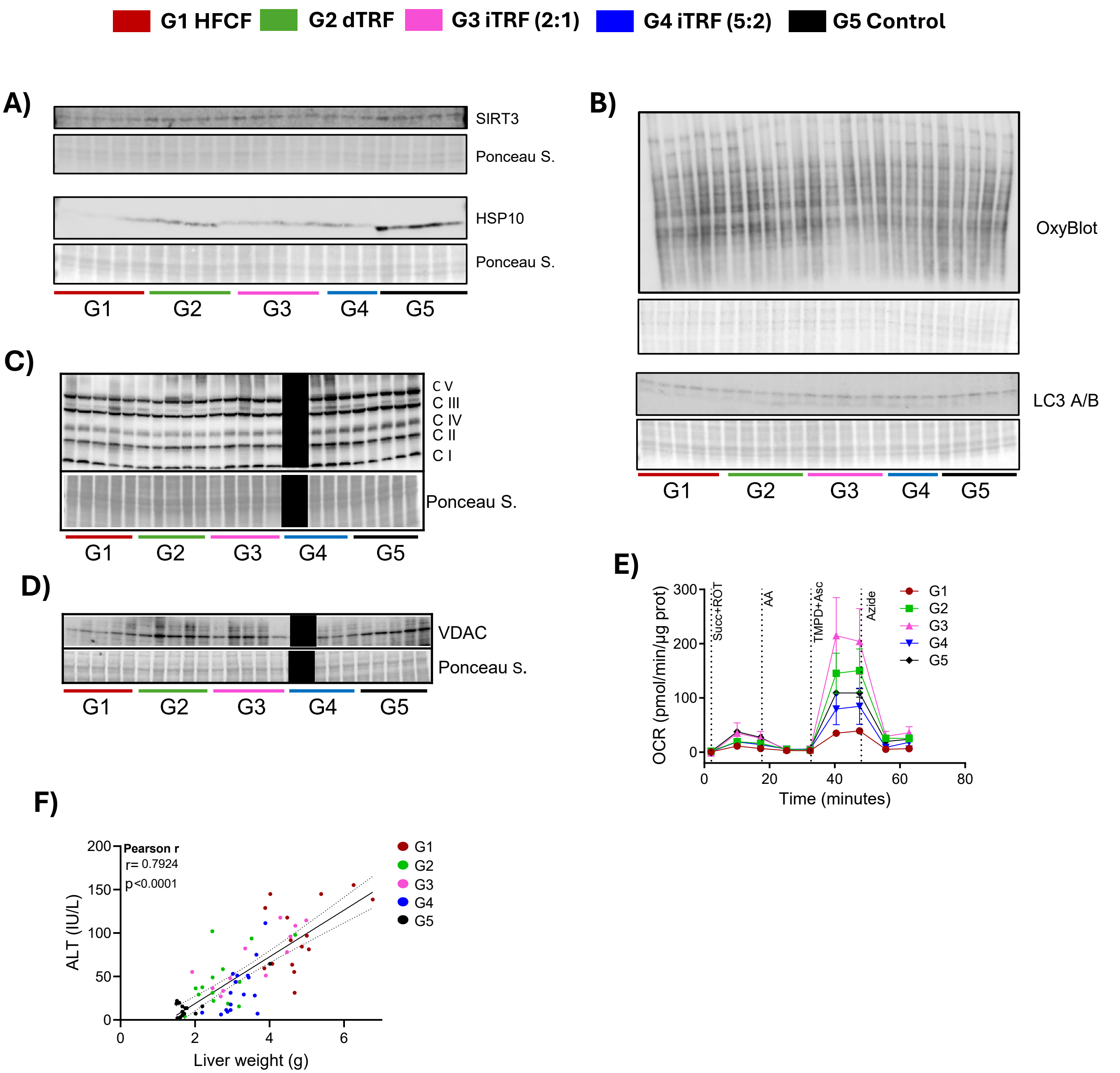

Supplemental Figure 6, related to Figures 3 and 4

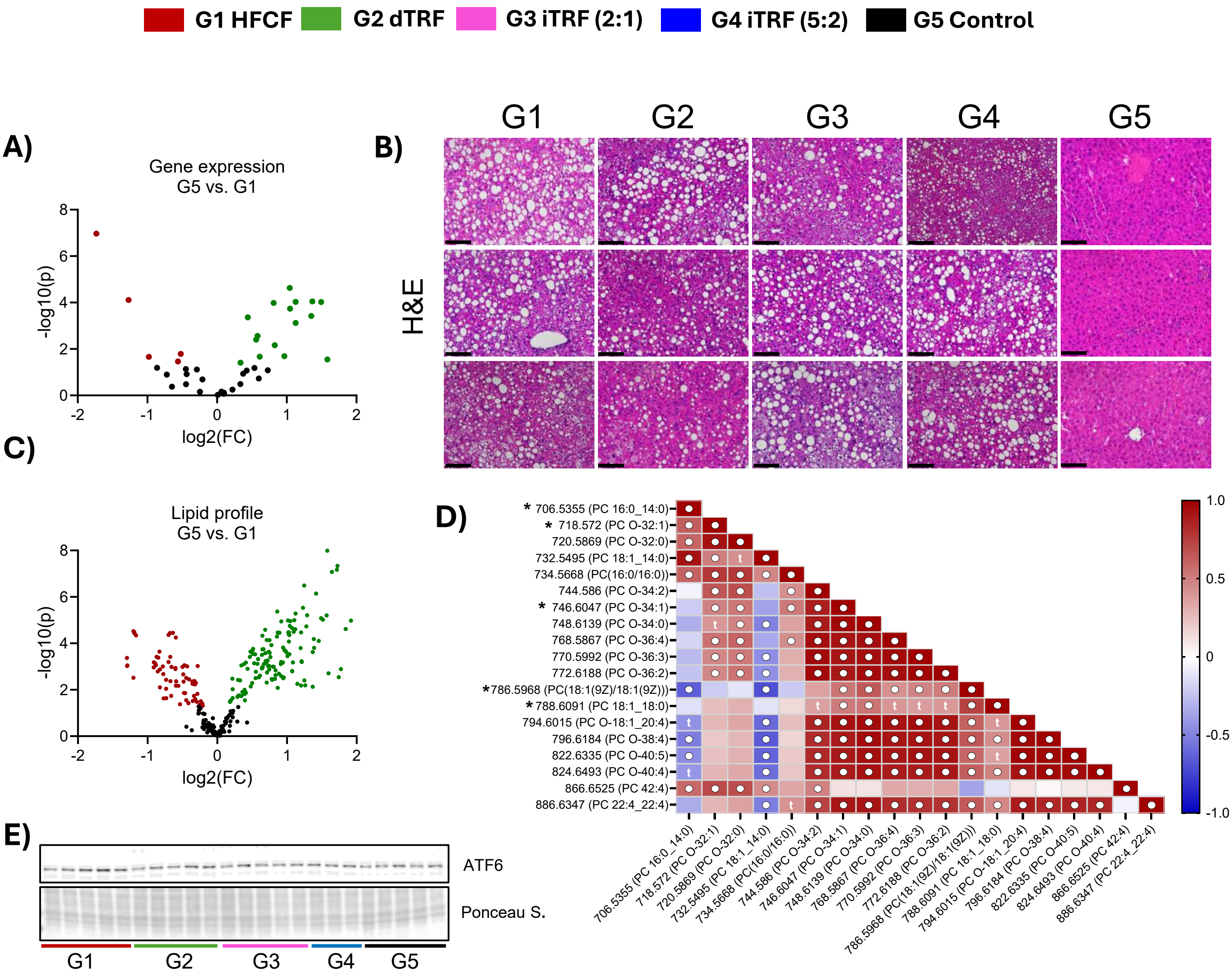

Supplement: Supplementary file 1 [file mmc1.pdf]
