# Supplementary material for: Intermittent time-restricted feeding recapitulates the physiological benefits of daily time-restricted feeding in male mice with chronic metabolic disease
Source: Mol Metab. 2026 Aug 25;113:102432. doi: 10.1016/j.molmet.2026.102432 (PMC13587810; doi:10.1016/j.molmet.2026.102432)
Supplement: Supplementary file 2 [file mmc3.pdf]

**Supplementary table 1** – Antibodies used to quantify specific proteins by Western Blot.

| Primary Antibodies | Dilution | Reference | Secondary Antibodies | Dilution | Reference |
|---|---|---|---|---|---|
| **ATF6** | 1:1000 | Santa Cruz sc-22799 | Anti-Mouse IgG | 1:5000 | Thermo A-21057 |
| **HSP10** | 1:1000 | Cell Signaling 53668 | Anti-Rabbit IgG | 1:5000 | Thermo A-10043 |
| **LC3A/B** | 1:1000 | Cell Signaling 4108S | Anti-Rabbit IgG | 1:5000 | Thermo A-10043 |
| **SIRT3** | 1:1000 | Cell Signaling 5490S | Anti-Rabbit IgG | 1:5000 | Thermo A-10043 |
| **OxyBlot** | 1:150 | Kit 1° Antibody stock (90451) | Kit 2° Antibody stock (90452) | 1:300 | Sigma S7150 |
| **OxPhos rodent WB antibody cocktail*** | 1:4000 | Life technologies 458099 | Anti-Mouse IgG | 1:5000 | Sigma A-9044 |
| **VDAC** | 1:1000 | SCBT SC-98708 | Anti-Rabbit IgG | 1:5000 | Sigma A-9169 |

* OxPhos rodent WB antibody cocktail:

Complex I subunit NDUFB8 (NADH dehydrogenase (ubiquinone) 1 beta subcomplex subunit 8)

Complex II subunit SDHB (Succinate dehydrogenase (ubiquinone) iron-sulfur subunit)

Complex III subunit UQCRC2 (Cytochrome b-c1 complex subunit)

Complex IV subunit MTCO1 (mitochondrially encoded cytochrome c oxidase I)

Complex V subunit ATP5A (Complex V alpha subunit)

**Supplementary Table 2** - Specific set of primers used for the amplification mouse transcripts by qPCR using the Standard BioTools platform. *Ppia*, *Actinb* and *Hprt* gene expression were used to generate a normalization factor for all other genes.

| Gene | Primer Sequence Sn | Primer Sequence As | NCBI Reference Sequence |
|---|---|---|---|
| ***Ppia*** | TGGTCTTTGGGAAGGTGAAAG | TGTCCACAGTCGGAAATGGT | NM_008907.1 |
| ***Actinb*** | CTGGGACGACATGGAGAAGA | ACCAGAGGCATACAGGGACA | NM_007393.3 |
| ***Hprt*** | CAAACTTTGCTTTCCCTGGT | TCTGGCCTGTATCCAACACTTC | NM_013556 |
| ***Acc1*** | ATCCTGCGAACCTGGATTCT | CCCACCAGAGAAACCTCTCC | NM_133360.2 |
| ***Acly*** | CCTTGAAGGAAGCAGGAGTG | GGTCATGAATGAGGCAGGTT | NM_001199296.1 |
| ***Acsl1*** | GAGGGTGAGGTGTGTGTGAAA | CCGTGTGTAACCAGCCATCT | NM_007981 |
| ***ApoB*** | AGCCCAGCACTGACTGACTT | GAAGCCTTGGGCACATTG | NM_009693.2 |
| ***Atgl*** | CTGATGACCACCCTTTCCAA | TGCTACCCGTCTGCTCTTTC | NM_001163689 |
| ***Cd36*** | GGAGCCATCTTTGAGCCTTC | TGGATCTTTGTAACCCCACAAG | NM_001159558 |
| ***Chrebp A*** | CGACACTCACCCACCTCTTC | TTGTTCAGCCGGATCTTGTC | NM_021455.4 |
| ***Chrebp B*** | TCTGCAGATCGCGTGGAG | CTTGTCCCGGCATAGCAAC | XM_006504483.2 |
| ***Cidea*** | GCAGCCTGCAGGAACTTATC | TCATGAAATGCGTGTTGTCC | NM_007702.2 |
| ***Cpt1*** | ACAACAACGGCAGAGCAGAG | GGACACCACATAGAGGCAGAAG | NM_013495 |
| ***Cyp4a10*** | CCACTGATTCTGTTGTGGAGC | CATTAGAAGAGAGGGGATGAGGA | NM_010011.3 |
| ***Dgat1*** | AGCTGTGGCCTTACTGGTTG | AGCAGCCCCACTGACCTT | NM_010046.2 |
| ***Elovl6*** | TCCAATGGATGCAGGAAAAC | AAGAGTCAGCGACCAGAGCA | NM_130450.2 |
| ***Fabp1*** | TTGCCACCATGAACTTCTCC | CCCTTGATGTCCTTCCCTTT | NM_017399.4 |
| ***Fabp4*** | AAGAAGTGGGAGTGGGCTTT | GCTCTTCACCTTCCTGTCGT | NM_024406.2 |
| ***Fasn*** | TGAGCACACTGCTGGTGAAC | CAGGTTCGGAATGCTATCCA | NM_007988.3 |
| ***FoxO1*** | GAAAATCACCCCGGAGAAGAG | ACTCGCAGGCCACTTAGAAAA | NM_019739.3 |
| ***Fsp27*** | AAGATGGCACAATCGTGGAG | TTAGTTGGCTTCTGGGAAAGG | NM_178373.3 |
| ***Fxr*** | GGAGGCCATGTTTCTTCGTT | AGCGTACTCCTCCTGAGTCATTT | NM_001163504.1 |
| ***Gpat1*** | GGTGAGGAGCAGCGAGATT | GGACAAAGATGGCAGCAGAG | NM_008149 |
| ***Hl*** | TTTTCCTGGTGTTCTGCATCT | CAGGCGATCGTTTTCATCTT | NM_008280.2 |
| ***Hmgcr*** | AGCACAGAATGTGGGGAGTT | TGCGTTTTCTCCAGGATTG | NM_008255.2 |
| ***Hmgcs2*** | TCAGGGGTCTAAAGCTGGAA | TAAGCCTGAGCCGTAGGAGA | NM_008256.44 |
| ***HNF4a*** | ACATTCGGGCAAAGAAGATTG | ACCTGGTCATCCAGAAGGAGTT | NM_008261.2 |
| ***Hsl*** | GCACTACAAACGCAACGAGA | CAGAGACGACAGCACCTCAA | NM_010719.5 |
| ***Ldlr*** | TGTCACCTGTCAGTCCAATCAA | TCAGAGCCATCTAGGCAATCTC | NM_001252658.1 |
| ***Lpl*** | TATCCCAATGGAGGCACTTTC | TGCTGGGGTTTTCTTCATTC | NM_008509.2 |
| ***Lrp1*** | ACACACGCCAACTGTACCAA | TGACATTCGGGTCACAAACA | NM_008512.2 |
| ***Me1*** | TATCCATGACAAAGGGCACA | TACAGCCAAGGTCTCCCAAG | NM_001198933.1 |
| ***Mogat1*** | TCTGGTTCTGTTTCCCGTTG | ACATTGCCACCTCCATCCTT | NM_026713.3 |
| ***Mogat2*** | TGTGAAAACTTGGAAATCGACA | CAGTCTCCAGCATGAAAAATCC | NM_146035.2 |
| ***Mttp*** | CAGTGGATGCCTCTTTTGTG | GTCTCGAATTGCCTGAGTGG | NM_001163457.1 |
| ***Mvk*** | CCTCCATTGACGCAATATCC | CTGGTTCATGTCTATCAGCTCTTC | NM_023556.2 |
| ***Pgc1a*** | TTCCCGATCACCATATTCCA | TTCATCCCTCTTGAGCCTTTC | NM_008904.2 |
| ***PPARa*** | GGGAAAGACCAGCAACAACC | GCAGTGGAAGAATCGGACCT | NM_011144 |
| ***PPARg*** | AGACCACTCGCATTCCTTTG | CCTGTTGTAGAGCTGGGTCTTT | NM_001127330.1 |
| ***Scd1*** | ATCGCCCCTACGACAAGAAC | GTTGATGTGCCAGCGGTACT | NM_009127.4 |
| ***Srb1*** | ATGCCCCAGGTTCTTCACTAC | GAGCCCTTTTTACTACCACTCCA | NM_016741.2 |
| ***Srebp1c*** | GGAGCCATGGATTGCACATT | GGCCCGGGAAGTCACTGT | XM_006532716.1 |
| ***Srebp2*** | TGGGAGAGTTCCCTGATTTG | TAATGGGACCTGGCTGAATG | NM_033218.1 |
| ***Synd*** | AAGGAAGTGCTGGGAGGTGT | CTCCTGCTTGGTGGGTTTCT | NM_011519.2 |
| ***Ucp2*** | TCTGGATACCGCCAAGGTC | ATTGTAGAGGCTGCGTGGAC | NM_011671 |
| ***Vldlr*** | GTGCAAGGCAGTAGGCAAAG | GCTGAGATCAGCCCAAAACA | NM_013703.2 |

**Supplementary Table 4** – Whole body Fat mass, Lean mass, and Lean mass to Fat mass ratio change from the intervention (week 23) until the end of the study. Data presented as Mean ± SEM (n=18-19). Letters in parentheses indicate significant differences (a, b and c). Groups that share the same letter are not statistically different. HFHC - animals fed *ad libitum* with a high fat diet with added cholesterol and 10% fructose in drinking water; dTRF - daily Time Restriction Feeding; iTRF 2:1 and iTRF 5:2 - intermittent TRF; Chow – standard maintenance diet. For further information about time feeding patterns see material and methos section.

| | **HFCF** | **dTRF** | **iTRF 2:1** | **iTRF 5:2** | **Chow** |
|---|---|---|---|---|---|
| **% Fat mass change from week 23** | 6.0 ± 2.5 (a) | -16.9 ± 6.2 (b) | -10.1 ± 4.7 (a,b) | -10.0 ± 6.0 (b) | -72.9 ± 2.1 (c) |
| **% Lean mass change from week 23** | -2.8 ± 2.0 (a) | 12.4 ± 3.8 (b) | 6.6 ± 3.0 (a,b) | 12.7 ± 3.2 (b) | 55.1 ± 3.0 (c) |
| **Lean mass/ Fat mass ratio change from week 23** | -11.2 ± 1.9 (a) | 98.0 ± 53.0 (b) | 24.1 ± 8.4 (b) | 72.6 ± 28.3 (b) | 550.1 ± 66.0 (c) |
