# Supplementary material for: Intermittent time-restricted feeding recapitulates the physiological benefits of daily time-restricted feeding in male mice with chronic metabolic disease
Source: Mol Metab. 2026 Aug 25;113:102432. doi: 10.1016/j.molmet.2026.102432 (PMC13587810; doi:10.1016/j.molmet.2026.102432)
Supplement: Supplementary file 3 [file mmc2.docx]

**Supplemental Figure 1 - Chronic metabolic imbalance induced by HFHC was significantly impacted by SD or TRF interventions. A)** Schematic representation of the daily feeding and light-dark cycles, highlighting the timing of sacrifice and tissue collection for the different experimental groups. **B-E)** Body weight (BW, **B**), whole body fat mass and lean mass percentages (**C** and **D**) and lean mass to fat mass ratio (**E**) from week 0 (set at 100%) to week 23. **F-I)** SD diet effect on G5 mice group on Body weight (BW, **F**), whole body fat mass and lean mass percentages (**G** and **H**) and lean mass to fat mass ratio (**I**) from week 23 (set at 100%) to week 37. Data are presented as the mean ± SEM. For panels A-H a t-test or Mann Whitney test (if one or more groups did not follow normal distribution assessed by Shapiro-Wilk test) comparing HFCF group at week0 (set at 100%) and 23 of the study or comparing SD control group between week 23 (set at 100%) with week 37. On A-H, asterisks indicate significant difference (**** p< 0.0001).

**Supplemental Figure 2 – Food intake and Body composition, independently of iTRF pattern. A)** Average daily food intake during 60 days of intervention. **B)** Average daily food intake during 60 days of intervention considering only *ad libitum* days. **C-D)** Daily food intake over the all-day experimental period. Comparison of daily energy consumption between experimental groups G1 and G3 (C) and Comparison of daily energy consumption between experimental groups G1 and G4 (D). **E)** Feed efficiency represented by body weight change relative to total energy intake. **F)** Cumulative food intake over the experimental period. Progression and endpoint comparison of cumulative food consumption among the experimental groups (G1–G5). Left Panel: Longitudinal progression of cumulative food intake per mouse (g/mouse) plotted daily up to final day under the iTRF (intermittent Time-Restricted Feeding) protocol. Right Panel: Represents the final cumulative food intake at the conclusion of the study (indicated by the boxed area on final day). **G-I)** Adipose tissue depots: visceral white adipose tissue (vWAT, **G**), subcutaneous white adipose tissue (sWAT, **H**) and brown adipose tissue (BAT, **I**). Data are presented as the mean ± SEM. Columns that do not share a common letter (a, b, c) are statistically different (p < 0.05).

**Supplemental Figure 3 – Correlation of Plasma Leptin with Visceral, Subcutaneous, and Brown Adipose Tissues and Independent Effects of TRF Pattern on Glycemic Control and Metabolic Parameters. A-C)** Correlation between plasma leptin levels and visceral (vWAT, **A**), subcutaneous (sWAT, **B**) and brown (BAT, **C**) adipose tissues. **D-E)** Glucose plasma levels in response to an intraperitoneal injection of insulin (insulin tolerance test, ITT, **D**) or glucose (glucose tolerance test, GTT, **E**). Panel A-C, linear regression with Pearson correlation value (r) and p-value. Data are presented as mean ± SEM.

**Supplemental Figure 4 - TRF interventions, independently of the pattern, positively modulated respiratory exchange ratio** (RER)**. A)** RER during light cycle during TRF period. **B)** RER average during the first 5h of the dark cycle after food reintroduction after a fasting period. For panels B a t-test comparing HFCF group (set at 100%) was performed (after confirming normal distribution accessed by Shapiro-Wilk test).

**Supplemental Figure 5 – Western Blots images and mitochondrial respiration. A-D)** SIRT3 and HSP10 (A), Oxyblot and LC3A/B (B), Mitochondrial respiratory complexes I-V (C), and VDAC (D). **E)** Mitochondrial respiration capacity considering individual interventions. **H)** Correlation between ALT plasma levels and liver weight. Linear regression with Pearson correlation value (r) and p-value (panel F). Succ. – succinate; ROT – rotenone; AA – antimycin A; TMPD – N,N,N',N'-tetramethyl-p-phenylenediamine.

**Supplemental Figure 6 – Hepatic Lipid-Related Gene Expression, Lipidomic Remodeling, and ER Stress Markers in Response to Diet and TRF Conditions. A)** Volcano plot representing the 43 lipid related genes expression levels (SD/HFCF group, red dots indicate genes significantly downregulated on chow diet control group and green dots indicate genes significantly upregulated on chow diet control group, p<0.05). **B)** Representative histological images of livers Hematoxylin and Eosin staining. **C)** Volcano plot representing all detected lipids from lipidomics analysis (SD/HFCF group, red dots indicate genes significantly downregulated on chow diet control group and green dots indicate genes significantly upregulated on chow diet control group, p<0.05 with FC of 1.1 or more). **D)** Lipid z-score calculated by the sum of z-scores of the 25 lipides defining clusters A, B and C from panel B (Figure 5B) independently of the daily TRF pattern. **E)** ATF6 western blot images. A and D, data represented as Fold Change [log2(FC), SD/HFCF] and t-test p-value (red and green dots (A) and red and green dots (F) indicate genes/lipids with FC of 1.1 or more and p-value<0.05). On panel D, data is represented as Pearson correlation value heatmap with white dots indicating statistical difference (p < 0.05) or a “t” for a tendency when p-value is between 0.05 and 0.1.
